# Development of a novel glycolysis-related genes signature for isocitrate dehydrogenase 1-associated glioblastoma multiforme

**DOI:** 10.3389/fimmu.2022.950917

**Published:** 2022-10-28

**Authors:** Xiaomin Cai, Zheng Chen, Caiquan Huang, Jie Shen, Wenxian Zeng, Shuang Feng, Yu Liu, Shiting Li, Ming Chen

**Affiliations:** ^1^ Department of Neurosurgery, Xinhua Hospital, Shanghai Jiaotong University School of Medicine, Shanghai, China; ^2^ Department of Neurosurgery, Sichuan Provincial People’s Hospital, University of Electronic Science and Technology of China, Chengdu, China; ^3^ Department of Neurosurgery, Zhujiang Hospital, Southern Medical University, Guangzhou, China; ^4^ Department of Encephalopathy, The Third Affiliated Hospital of Nanjing University of Chinese Medicine, Nanjing, China; ^5^ Department of Neurosurgery, Shanghai Children’s Hospital, Shanghai Jiaotong University, Shanghai, China; ^6^ Department of Neurosurgery, Xinhua Hospital, Shanghai Jiaotong University School of Medicine, the Cranial Nerve Disease Center of Shanghai Jiaotong University, Shanghai, China

**Keywords:** glioblastoma multiforme, glycolysis, prognosis, isocitrate dehydrogenase 1, immunity

## Abstract

**Background:**

The significant difference in prognosis between IDH1 wild-type and IDH1 mutant glioblastoma multiforme (GBM) may be attributed to their metabolic discrepancies. Hence, we try to construct a prognostic signature based on glycolysis-related genes (GRGs) for IDH1-associated GBM and further investigate its relationships with immunity.

**Methods:**

Differentially expressed GRGs between IDH1 wild-type and IDH1 mutant GBM were screened based on the TCGA database and the Molecular Signature Database (MSigDB). Consensus Cluster Plus analysis and KEGG pathway analyses were used to establish a new GRGs set. WGCNA, univariate Cox, and LASSO regression analyses were then performed to construct the prognostic signature. Then, we evaluated association of the prognostic signature with patients’ survival, clinical characteristics, tumor immunogenicity, immune infiltration, and validated one hub gene.

**Results:**

956 differentially expressed genes (DEGs) between IDH1 wild-type and mutant GBM were screened out and six key prognostically related GRGs were rigorously selected to construct a prognostic signature. Further evaluation and validation showed that the signature independently predicted GBM patients’ prognosis with moderate accuracy. In addition, the prognostic signature was also significantly correlated with clinical traits (sex and MGMT promoter status), tumor immunogenicity (mRNAsi, EREG-mRNAsi and HRD-TAI), and immune infiltration (stemness index, immune cells infiltration, immune score, and gene mutation). Among six key prognostically related GRGs, CLEC5A was selected and validated to potentially play oncogenic roles in GBM.

**Conclusion:**

Construction of GRGs prognostic signature and identification of close correlation between the signature and immune landscape would suggest its potential applicability in immunotherapy of GBM in the future.

## Introduction

Glioblastoma multiforme (GBM) is the most malignant brain tumor in the central nervous system (CNS) (1, 2). Currently, the most effective treatment of GBM is gross total surgical resection and standard postoperative chemoradiotherapy. However, this treatment still delivers an insufficient therapeutic effect (3, 4). Isocitrate dehydrogenase 1 (IDH1) is a critical metabolic enzyme in the Krebs cycle (5). Importantly for the diagnosis of GBM, the determination of IDH1 mutation status has been included, resulting in distinct subgroups, namely, IDH1 mutant type (IDH1 MUT) and IDH1 wild type (IDH1 WT), since the 2016 WHO classification of CNS tumors was released (6). Numerous studies reported that IDH1 MUT GBM patients have better prognosis than IDH1 WT patients, but the specific mechanisms at a molecular level are still unknown. GBM arises in a hypoxic environment, being forced to modify its metabolic pathways to obtain nutrients (7–9). Altered cellular metabolism is a relevant hallmark of GBM (10). As a key enzyme in the Krebs cycle, IDH1 itself plays an important role in the metabolism of GBM (11). Thus, the difference in prognosis between IDH1 MUT and WT patients may be closely related to the metabolic difference between these two subgroups of GBM cells. Glycolysis is the metabolic pathway by which glucose is broken down into two molecules of pyruvate, while producing energy in the form of adenosine triphosphate (ATP) and nicotinamide adenine dinucleotide (NADH) (12). One of the best-known alterations in GBM cell metabolism is the capacity for aerobic glycolysis (13). So, this study will first try to find the reasons for the differences in prognosis by analyzing the DEGs related to glycolysis between IDH1 MUT and WT GBM patients.

In addition, aerobic glycolysis can promote apoptosis of GBM cells, induce GBM cells to differentiate into astrocytes, and destroy the immune microenvironment of tumors (14, 15). It was reported that 2-hydroxyglutarate (2-HG), which is generated by IDH1 MUT GBM cell’s glycolysis, could influence the tumor microenvironment and pH value and further suppress the action of immune cells (16). This means that glycolysis is closely related to GBM patients’ prognosis and therapeutic effect of immune treatment, especially in IDH1 MUT patients. Because the routine treatment strategy for GBM patients is not fully, some new methods, such as immune therapy, are applied in clinical practice.

Accumulating evidence has revealed that GRGs were differentially expressed in a number of malignant tumors and played critical roles in tumor initiation and development. For instance, Kimberly et al (17). reported that high expression of GRGs hexokinase 2 (HK2) and pyruvate kinase M2 (PKM2) was significantly associated with an increased risk for GBM formation and predicted a dismal outcome for GBM patients. Moreover, inhibitors targeting HK2 could be utilized to selectively kill cancer cells (18). To the best of our knowledge, more and more studies focusing on the relationships between glycolysis and GBM occurrence and development have received considerable attention in recent years, and underlying mechanisms of increased glycolytic activity in GBM has already been determined. However, there is currently no glycolysis-related prognostic model for GBM. Therefore, the present study aims to explore the possible reason for different prognosis between IDH1 MUT and WT GBM patients at basic research level and provide a prognostic prediction model and theoretical basis for new potential adjuvant postoperative treatment methods such as immune therapy in the future.

## Materials and methods

### Data preparation and collection

See **Appendix S1** for details.

### Differential expression and functional enrichment analysis

Limma R package and ClusterProfiler R package were used in screening and analyzing of differentially expressed genes (DEGs) between IDH1 MUT and WT GBM samples. See **Appendix S1** for details.

### Cluster analysis

Consensus Cluster Plus R package was used for subgroup cluster analysis based on the differential expression profiling of glycolysis-related genes. The cluster distance was “euclidean” and the cluster method was “km”. This analysis was repeated 100 times so as to ensure the stability of subgroup classification.

### Co-expression modules construction and hub genes identification

WGCNA was used to construct co-expression modules. Hub genes were identified through protein–protein interaction (PPI) network. See **Appendix S1** for details.

### Construction and validation of prognostic model

The hazard ratio (HR) and prognostic significance of differential expressed genes were determined by univariate Cox regression analysis, and the genes with *p*-value <0.05 were selected as prognosis-related genes. The least absolute shrinkage and selection operator (LASSO) regression was analyzed using “glmnet” R package to further screen prognostic factors. Detailed descriptions of calculation of risk score are provided in **Appendix S1**.

### Annotation of the immune infiltration microenvironment

The “estimate” R package was utilized to evaluate immune infiltration microenvironment. See **Appendix S1** for details.

### Prediction of immunotherapy response

See **Appendix S1** for details.

### Tissue samples and cell culture

Six IDH1 wild-type and IDH1 mutant GBMs were all obtained from the Department of Neurosurgery, Xinhua Hospital, affiliated with Shanghai Jiaotong University School of Medicine from November 2017 to July 2021. The histopathological features of these specimens were identified by two neuropathologists in accordance with the WHO criteria. Clinical information and molecular features of those GBM patients was shown in **Table S1**. The study protocol was reviewed and approved by the Human Ethics Committee of Xinhua Hospital and it conformed to the provisions of the Declaration of Helsinki. All patients had signed their written informed consent. Detailed method of cell culture is shown in **Appendix S1**.

### Lentiviral and plasmid transfection

See **Appendix S1** for details on lentiviral and plasmid construction, and transfection.

### Western blotting

Western blotting was performed as described previously (19). Detailed descriptions of the methods are provided in **Appendix S1**. Uncropped images of Western blotting are provided in **Figure S9**.

### Cell proliferation assay

Detailed methods are provided in **Appendix S1**.

### Transwell invasion assay and wound healing assay

Transwell invasion assay and wound healing assay were performed as previously described (20). See **Appendix S1** for details.

### Statistical analysis

Statistical analyses and visualization were mainly performed using R version 3.6.0 and GraphPad Prism version 9.0.0. Sources and versions of software and R packages used in this study were provided in **Table S6**. Wilcoxon test and Student’s t test were used to estimate the differences between two groups. Kruskal-Wallis analysis was used to estimate the differences between more than two groups. Two-sided *p-*value<0.05 was regarded as statistically significant. (ns: p>0.05, *: p ≤ 0.05, **: p ≤ 0.01, ***: p ≤ 0.001, ****: p ≤ 0.0001)

## Results

### Determination of a new glycolysis-related gene set based on DEGs

**Figure 1:**
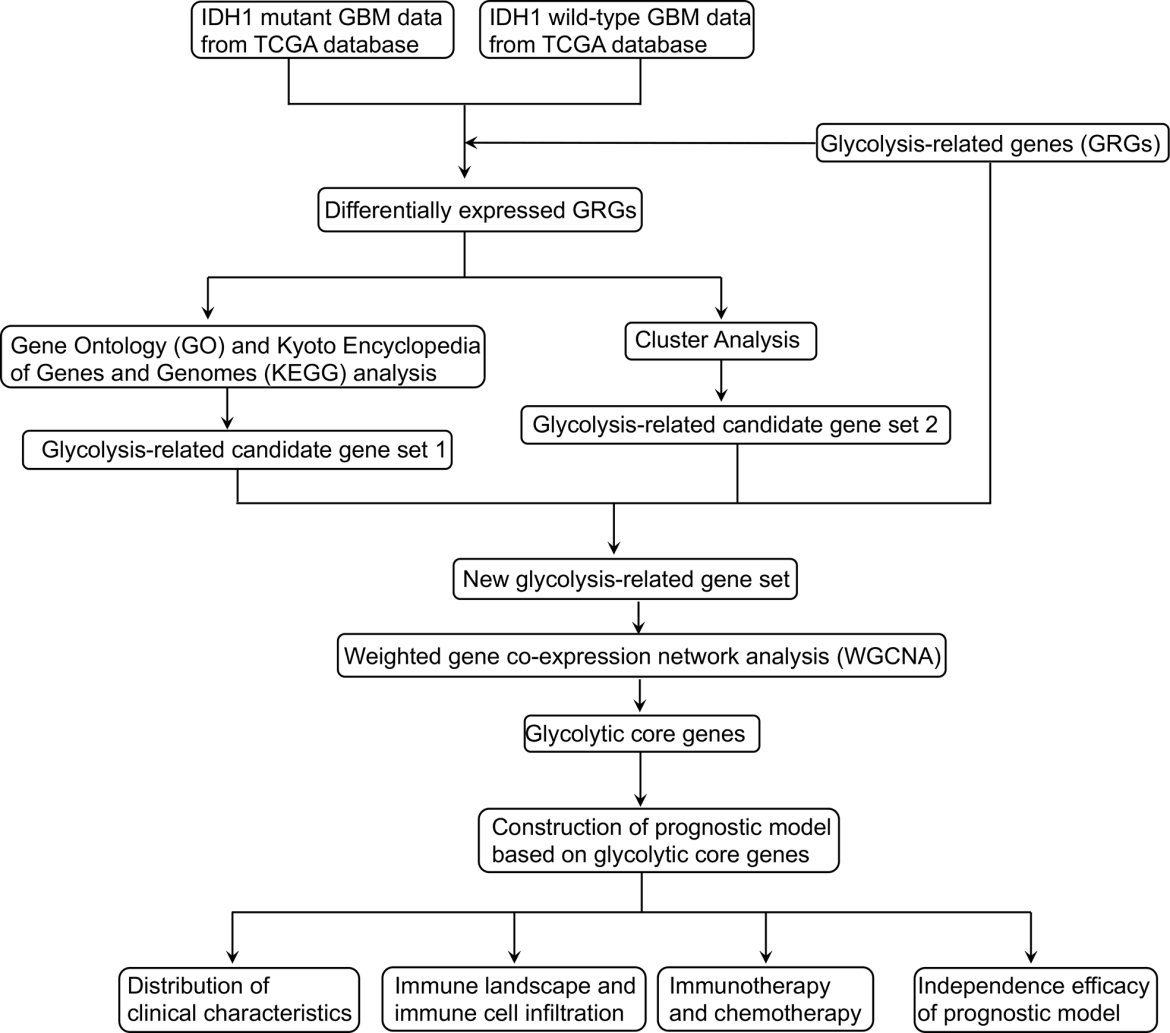
The flow diagram of this study.

**Figure 2:**
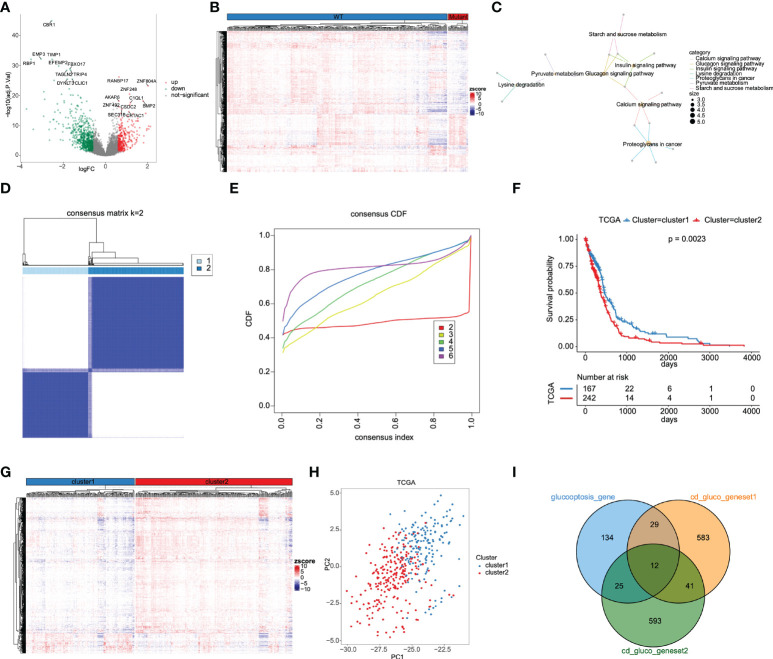
Determination of GRG set based on DEGs between IDH MUT and WT GBM. **(A)** The volcano plot for DEGs between IDH MUT and WT GBM from the TCGA database. **(B)** Heatmap of DEGs expression levels. **(C)** KEGG functional enrichment analysis of 37 differentially expressed GRGs. **(D)** 409 GBM samples were classified into two subtypes according to 37 differentially expressed GRGs, named cluster 1 and cluster 2. **(E)** Consensus cumulative distribution function. **(F)** Kaplan-Meier survival curves for cluster 1 and cluster 2 subtypes. **(G)** Heatmap of DEGs between cluster 1 and cluster 2 subtypes. **(H)** Cluster 1 and cluster 2 are clearly distinguished using principal component analysis (PCA). **(I)** Construction of a whole new GRG set (1417 genes).

The design of our study is shown in **Figure 1**. DEGs were screened and listed by “limma” R package according to difference multiple and significance threshold. A total of 409 GBM samples, including 375 IDH1 MUT and 34 IDH1 WT samples, from the TCGA database were used for analysis. Results showed that the expression of 310 genes was upregulated and the expression of 646 genes was downregulated (**Figures 2A, B**). Original GRG set, which includes 200 genes, was downloaded from the MSigDB (http://www.gsea-msigdb.org/gsea/index.jsp). The number of intersection genes between DEGs (956 genes) and GRG set (200 genes) was 37. KEGG analyses of the 37 intersecting GRGs were conducted by using “ClusterProfiler” R package. The results showed that these 37 genes are mainly involved in seven signaling pathways (**Figure 2C**). The “glycolysis-related candidate gene set 1” including 665 genes was then obtained after combining all the genes involved in the above-mentioned signaling pathways and removing redundancy. Cluster analysis was conducted based on the expression profiles of 37 intersecting GRGs. Subsequently, 409 GBM samples were divided into two different subtypes (**Figures 2D, E**). We named these two subtypes “cluster 1” (167 samples) and “cluster 2” (242 samples). To be noted, the prognosis of samples of these two kinds of subtypes was significantly different (**Figure 2F**). According to the difference in multiple and significance threshold, 671 DEGs between “cluster 1” and “cluster 2” were screened out by “Limma” R package (**Figure 2G**). Principal component analysis was used to compare the two subtypes. Results indicated that the principal components of DEGs can clearly distinguish the two subtypes (**Figure 2H**). Thus, we named these 671 DEGs “glycolysis-related candidate gene set 2”. Then, a whole new glycolysis-related gene set (1417 genes) was constructed as a union by combining original GRG set (200 genes), “glycolysis-related candidate gene set 1” (665 genes), and “glycolysis-related candidate gene set 2” (671 genes) (**Figure 2I**).

### Identification of the key modules and hub genes using WGCNA

**Figure 3:**
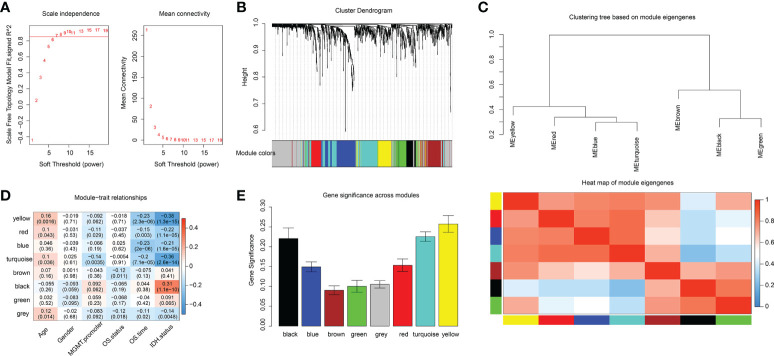
Identification of key modules associated with clinical traits of GBM based on the TCGA database by WGCNA. **(A)** Analysis of network topology for various soft-thresholding powers. **(B)** Clustering dendrograms of genes, with dissimilarities based on topological overlap, with assigned module colors. **(C)** The module eigengenes adjacency presented by hierarchical clustering (upper panel) and heat map (lower panel). The gene expression level of each module was represented with module eigengenes. **(D)** Module-trait relationships. Each row represents a module eigengene, each column corresponds to a clinical trait of GBM. The corresponding correlation coefficient and p-value are shown in each cell. **(E)** Distribution of average gene significance in the modules associated with IDH status of GBM.

**Figure 4:**
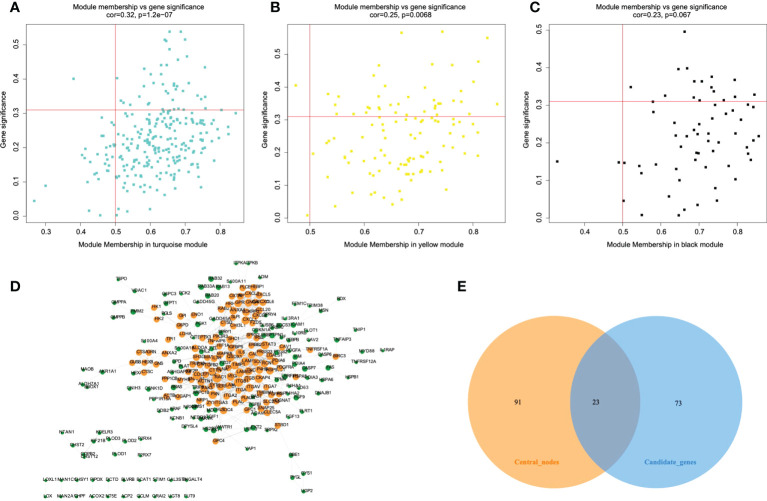
Protein-protein interaction (PPI) network construction and identification of hub genes. A-C. The scatterplot of gene significance vs. module membership in the key co-expression modules, presented with turquoise **(A)**, yellow **(B)**, and black **(C)** colors respectively. **(D)** Establishment of PPI network based on genes from key modules. Yellow color represents network core genes. **(E)** The Venn diagram shows the intersecting genes of module core genes and network core genes.

Among 1417 GRGs mentioned above, only 1281 genes can be found in the TCGA database. Thus, based on these 1281 genes, WGCNA was performed by R package. Results showed that the co-expression network conformed to the scale-free networks, and the correlation coefficient was greater than 0.8. In order to ensure that the network was scale-free, we chose the optimal β= 7 (scale independence >0.85) (**Figure 3A**). Next, we performed cluster analysis on the modules according to dynamic cutting method and then determined eight modules (**Figure 3B**). As shown in **Figure 3C**, we calculated module eigengenes which summarize the gene expression profile of each module and corresponding heat map of module eigengenes was also provided. Pearson correlation coefficient between each module and phenotypic characteristics of the sample was calculated. The numbers in each cell represented the correlation coefficient between the gene module and the phenotypic characteristics of the sample, and the numbers in brackets represented the significance p value (**Figure 3D**). Then, the gene significance (GS) value of each gene module was calculated. The greater the GS value was, the more relevant the module was to the IDH1 mutation status (**Figure 3E**). Hence, turquoise, yellow, and black were selected as the key modules. According to module membership (MM) > 0.5 and GS > 0.31, 96 module core genes were screened out from the three above-mentioned modules (**Figures 4A–C**). Protein-protein interaction (PPI) of genes in three modules was obtained based on the STRING database (https://www.string-db.org/). When the degree was greater than or equal to 5, 114 network core genes were acquired (**Figure 4D**). The intersection of module core genes and network core genes was taken, and the 23 genes in the intersection were used as glycolytic core genes for subsequent analysis (**Figure 4E**). In addition, we also constructed a glycolytic core gene-centered multi-factor regulatory network, which was composed of 55960 edges and 10873 nodes, including 9847 lncRNAs, 969 miRNAs, 23 mRNAs, and 34 transcription factors (**Figure S1**).

### Screening of overall survival–associated glycolytic core genes and construction of the glycolysis-related prognostic model

**Figure 5:**
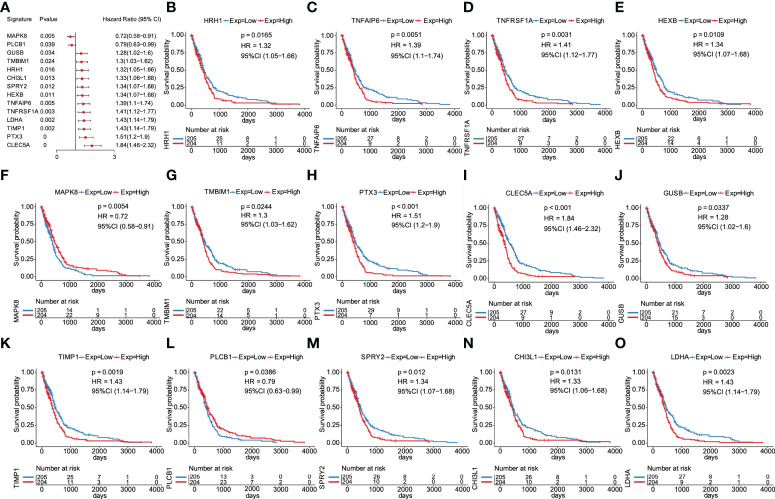
Screening of overall survival–associated glycolytic core genes. **(A)** 14 genes significantly associated with GBM patients’ OS among 23 glycolytic core genes. **(B)** Survival analysis of 14 genes for GBM patients in the TCGA database, including HRH1 **(B)**, TNFAIP6 **(C)**, TNFRSF1A **(D)**, HEXB **(E)**, MAPK8 **(F)**, **(G)**, PTX3 **(H)**, CLEC5A **(I)**, GUSB **(J)**, TIMP1 **(K)**, PLCB1 **(L)**, SPRY2 **(M)**, CHI3L1 **(N)**, and LDHA **(O)**.

The prognostic value of the 23 glycolytic core genes was defined by univariate Cox regression analysis (**Table S2**). In this analysis, genes were regarded as significant at p <0.05. We found that 14 genes were significantly associated with GBM patients’ overall survival (OS) (**Figure 5A**). The survival curves of 14 prognostic genes were shown in **Figures 5B–O**, which indicated that high expression of these genes all correlated with a poor outcome for GBM. Interestingly, GUSB and LDHA were included in the original glycolysis-related gene set (200 genes). Next, six key prognostic genes were further screened out by LASSO method (**Figures S2A–C**). By weighting the expression of these six genes with LASSO regression coefficient, a risk score model for predicting prognosis was established by the following algorithm (exp: expression value of gene for each patient). Risk score = (CLEC5A exp * 0.16) + (TNFAIP6 exp * 0.03) + (PLCB1 exp * (-0.055)) + (MAPK8 exp * (-0.28)) + (TMBIM1 exp * 0.017) + (LDHA exp * 0.012).

### Evaluation and validation of our established model

**Figure 6:**
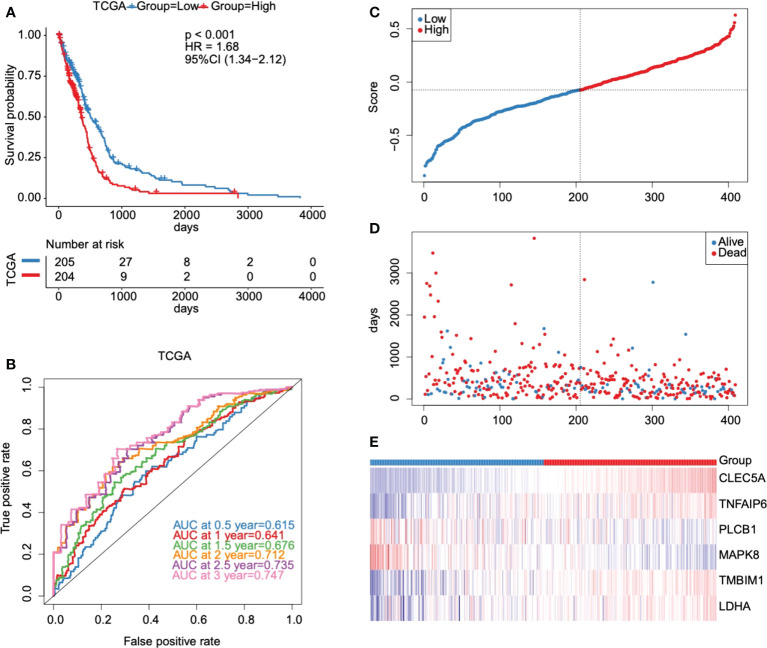
Evaluation and validation of the prognostic model in the TCGA database. **(A)** Kaplan-Meier survival curves for high- and low-risk groups stratified by the risk score model in the TCGA database. **(B)** ROC curves for predicting 0.5-year, 1-year, 1.5-year, 2-year, 2.5-year, and 3-year OS for GBM patients based on the risk score in the TCGA database. **(C–E)** Risk score distribution, GBM patients’ survival status, and six key prognostic glycolytic core genes expression heat map in the TCGA database.

According to the median cut-off value of risk score, the GBM patients were divided into high- and low-risk groups (n = 204/205). Kaplan-Meier curves were used to evaluate the performance of our established model in OS prediction. Results demonstrated that patients in the high-risk group had a worse prognosis compared with those in the low-risk group (**Figure 6A**). ROC was used to evaluate the prediction results of the model. The AUC of samples in 0.5 year, 1 year, 1.5 years, 2 years, 2.5 years, and 3 years reached 0.615, 0.641, 0.676, 0.712, 0.735, and 0.747 respectively (**Figure 6B**), indicating that the prediction effect of the model was satisfied. We also observed that the number of patients in the high-risk group and the number of dead patients grew when the risk scores increased (**Figures 6C, D**). As shown in **Figure 6E**, six key prognostic glycolytic core genes expression heat map in the TCGA database was also presented.

To determine whether the risk model had similar predictive values in different populations, two validation datasets were used, including CGGA_325 and CGGA_693 datasets. We found that samples in the high-risk group also showed a worse prognosis than those in the low-risk group (**Figure S3A**). The AUC values indicated a moderate prediction efficiency of the risk score model (**Figure S3B**). In addition, results showed that the number of patients in the high-risk group and the number of dead patients grew with the increase of the risk scores (**Figures S3C, D**). Expression heat map of six key prognostic glycolytic core genes in the CGGA_325 dataset was shown in **Figure S3E**. Further, similar results were obtained in the CGGA _693 dataset (**Figure S4**). Taken together, the risk score model we established can predict the prognosis of GBM well.

### Correlation between the prognostic model and clinical characteristics

**Figure 7:**
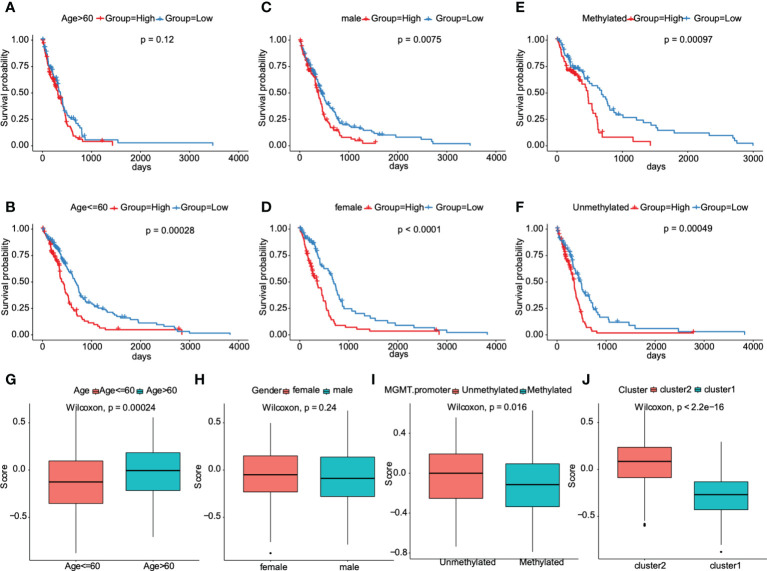
Association between the prognostic model and clinical traits for GBM patients. **A–F**. Subgroup analysis of GBM patients according to age> 60 or ≤60, sex, and the status of methylation. The prognostic model we constructed retained its stable prognostic value in multiple subgroups of GBM patients, including patients aged ≤60 years **(B)**, male or female patients **(C, D)**, and patients with/without methylation **(E, F)**. **G–J**. Patients with different clusters (cluster 1 or 2) and clinical features (including age> 60 or ≤60, with or without methylation) had different levels of risk scores, calculated according to the prognostic model.

The clinical characteristics of the TCGA dataset samples were grouped. Kaplan Meier survival analysis was used to evaluate the prognostic differences of high-risk and low-risk groups after samples with different clinical characteristics were grouped. We found that the risk score model had significant prognostic differences between groups with different gender (**Figures 7C, D**) and methylation status in O6-methylguanine-DNA methyltransferase (MGMT) promoter region (**Figures 7E, F**), and there were significant prognostic differences between groups in samples who were younger than or equal to 60 years (**Figures 7A, B**), indicating that the prediction ability of the risk score model was stable.

Furthermore, this prognostic model was verified in other cancers of TCGA dataset. The results showed that there were significant prognostic differences only in low-grade gliomas, indicating the heterogeneity of this model (**Figure S5**). We next tried to compare the distribution of risk scores in different characteristic groups. The results demonstrated that the risk scores of samples in the “cluster 2” group were significantly higher than those in “cluster 1” group, the risk scores of samples aged less than or equal to 60 years were significantly lower than those aged more than 60, and the risk scores of unmethylated samples in MGMT promoter region were significantly higher than those in methylated samples in MGMT promoter region (P<0.05, **Figures 7G, I, J**). And, no significant differences were found in risk score distribution between females and males (**Figure 7H**). All these results indicated that the prognostic model was markedly associated with clinical characteristics for GBM patients.

### Association between the risk score and immune landscape

**Figure 8:**
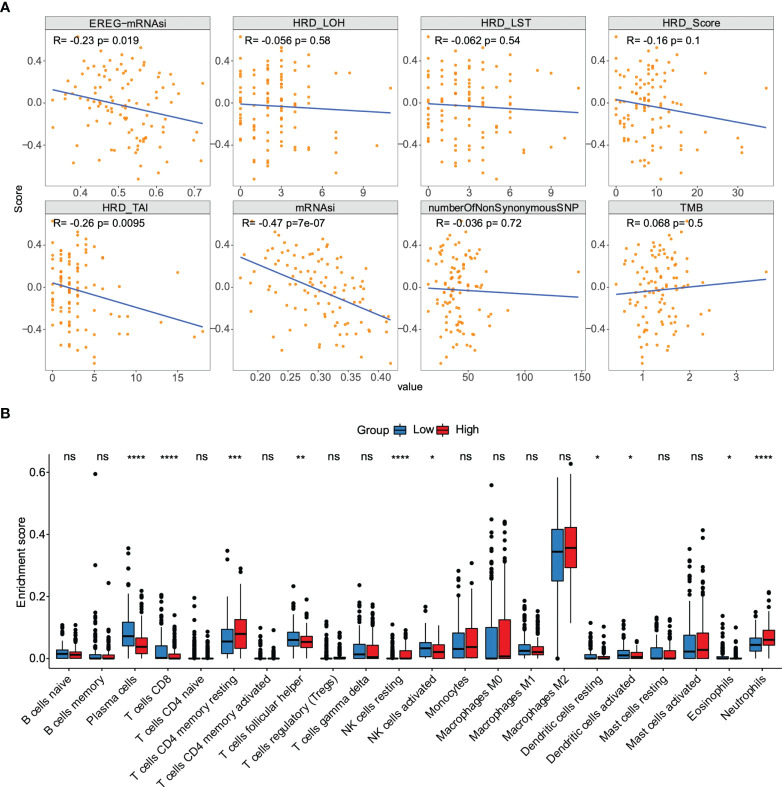
The association between the risk score and genetic characteristics and infiltration immune cells. **(A)** Correlation between risk score and some potential factors which determine tumor immunogenicity, including stemness index, chromosome instability level, homologous recombination defect, neoantigen load, and mutation load. **(B)** Compositions of infiltration immune cells between high‐ and low‐risk groups in the TCGA dataset. *p < 0.05, **p < 0.01, ***p < 0.001, ****p < 0.0001. ns, no significance.

**Figure 9:**
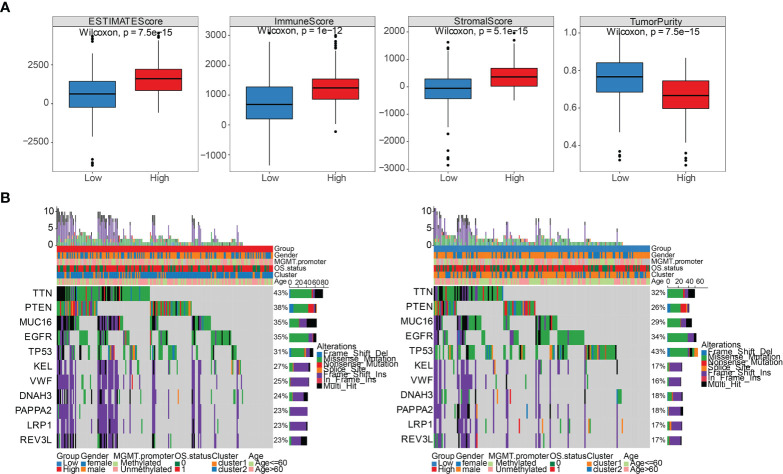
Comparison of immune stromal score and somatic cells mutation between high- and low-risk groups. **(A)** Differential distribution of estimate score, immune score, stromal score, and tumor purity between high- and low-risk groups. **(B)** 11 genes with mutation frequency in the top 20 simultaneously appeared in the high-risk group (left panel) and low-risk group (right panel).

We sought to further study the correlation between immune landscape and risk score. Our study results indicated that risk score significantly negatively correlated with the mRNA expression-based stemness index (mRNAsi), epigenetically regulated mRNAsi (EREG-mRNAsi), and homologous recombination deficiency-telomeric allelic imbalance score (HRD-TAI) (p<0.05, **Figure 8A**). The infiltration scores of 22 kinds of immune cells were calculated by CIBERSORT R package. The results showed that there were significant differences between high- and low-risk groups in enrichment scores of some kinds of immune cells, such as T cells CD4 memory resting, plasma cells, and T cells CD8 (p<0.05, **Figure 8B**). Further analyses showed that the high-risk group had a markedly higher estimate score, immune score, and stromal score than those in the low-risk group. And the tumor purity score in the high-risk group was lower (p<0.05, **Figure 9A**). Then, we compared the differences of somatic cells mutations between the two groups. Study results showed that 11 genes with mutation frequency in the top 20 simultaneously appeared in the high-risk (**Figure 9B**, left panel) and low-risk group (**Figure 9B**, right panel). In addition, we presented the distribution of copy number variation (CNV) frequency and discovered lower CNV frequency of the high-risk group than the low-risk group (**Figure S6**). Together, these results revealed that risk score correlated with immune landscape.

### Relationship between immunotherapy and chemotherapy and risk score

**Figure 10:**
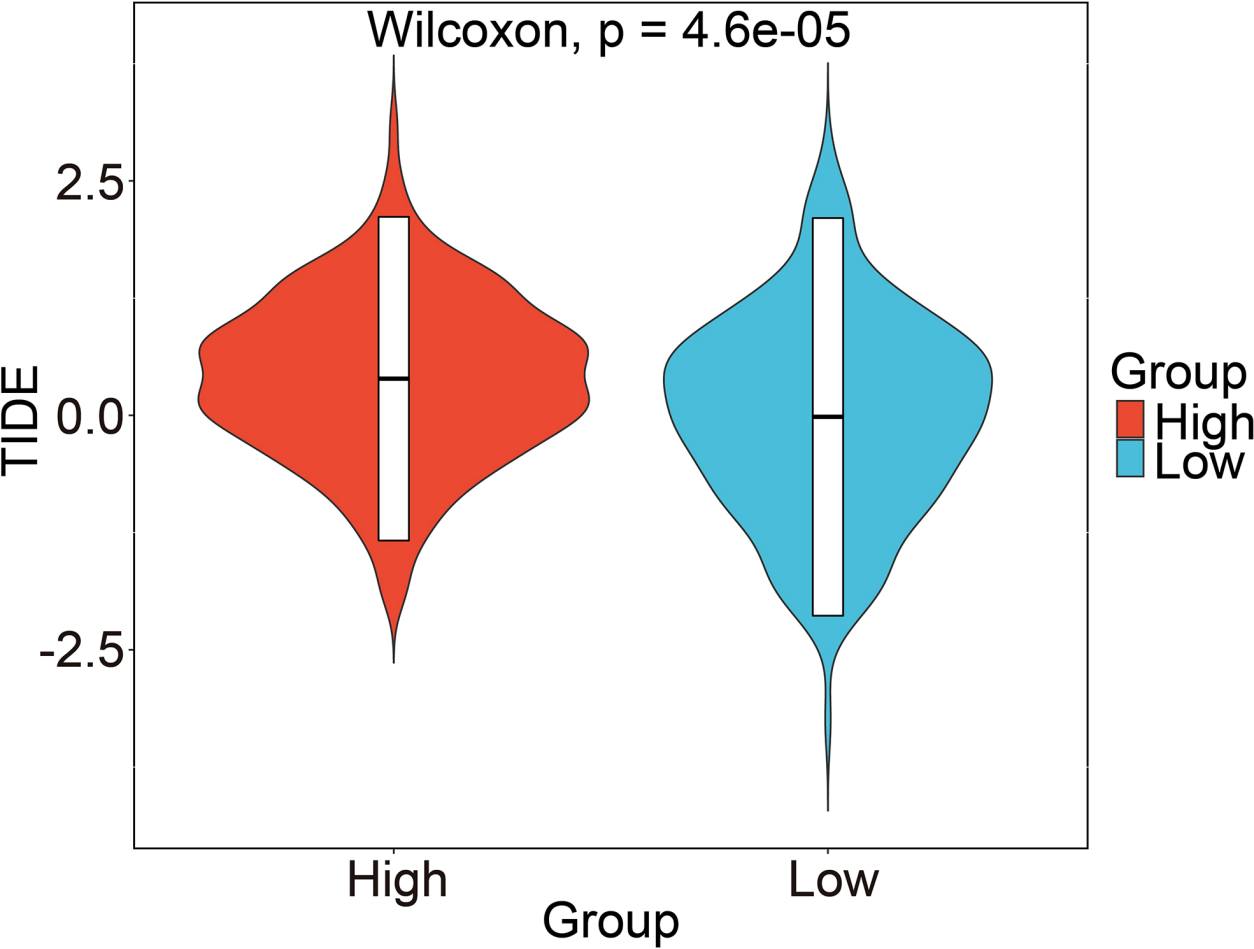
Prediction of risk score-related immune responses of immunotherapy. Prediction of risk score-related immune responses of immunotherapy. Distribution of risk score in TIDE scores by TIDE dataset.

The candidate IMvigor210 data set, which contains immunotherapy information of urothelial carcinoma, was selected to study whether the risk score can be used as a marker of immunotherapy response due to a shortage of six prognostic GRGs-related specific GBM immunotherapy data set. However, the results displayed no significant differences between high- and low-risk groups (**Figure S7A**). We analyzed the relative proportion of complete response/partial response (CR/PR) and stable disease/progressive disease (SD/PD) after patients received immunotherapy. Then, we found that the differences in distribution of immune response between high- and low-risk groups was not statistically significant (**Figure S7B**). And there were also no significant differences in the distribution of risk score in each immune response, including PR, PD, CR, and SD. (**Figure S7C**). Honestly, these results did not coincide with our expectations. Hence, we decided to assess the immune properties of risk score we established in IDH1-associated GBM by using the immunophenoscores of GBM patients from the Tumor Immune Dysfunction and Exclusion (TIDE) database. As shown in **Figure 10**, our results demonstrated that patients in the high-risk group had a higher TIDE score compared with those in low-risk group (p < 0.001), indicating a potential worse efficacy and more dismal outcome after acceptance of the immunotherapy treatment in the high-risk than low-risk group. And, we speculated that this result may be related to a higher probability of immune escape in the high-risk group. Taken together, our results demonstrated that the risk score model we constructed could be utilized to predict the potential clinical effects of immunotherapy for GBM patients. Next, we investigated whether the risk score can be used as a marker of chemotherapy response. Among the five commonly used chemotherapy drugs, there were significant differences in the drug resistance of samples in high- and low-risk groups to doxorubicin, vinblastine, and sorafenib (P<0.05, **Figure S8A**). In addition, Pearson correlation coefficient between each module and five commonly used chemotherapeutic drugs was also calculated. The numbers in each cell represented the correlation coefficient between the gene module and the chemotherapeutic drug, and the numbers in brackets represented the significance p value (**Figure S8B**).

### Independent prognostic value of prognostic model

**Figure 11:**
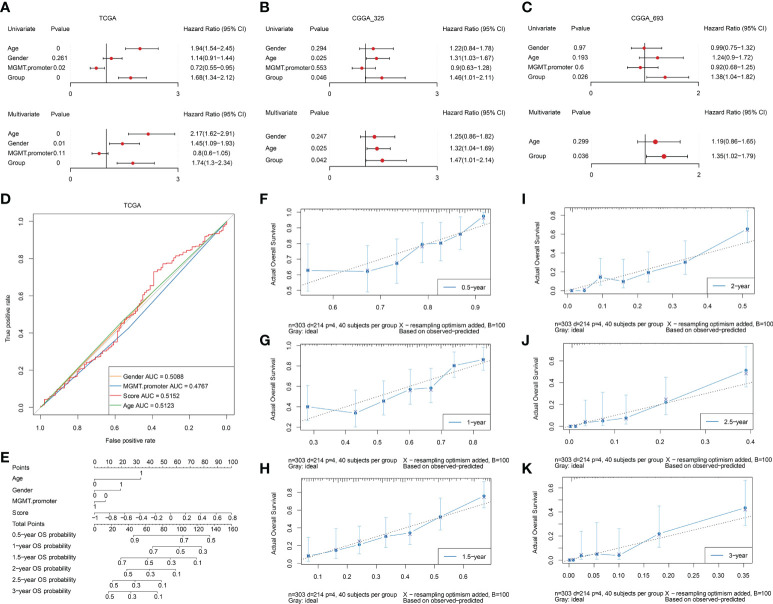
Assessment of independent efficacy of the prognostic model. A-C. Univariate (upper panel) and multivariate (lower panel) Cox regression analysis of the prognostic model and clinical features in the TCGA **(A)**, CGGA_325 **(B)**, and CGGA_693 **(C)** datasets, respectively. **(D)** Nomogram for predicting the prognosis of GBM patients, integrating three clinical features and the prognostic model we established. **(E)** Multi-ROC curves for predicting 0.5-year, 1-year, 1.5-year, 2-year, 2.5-year, and 3-year OS for GBM patients based on three clinical features and the prognostic model. F-K. The calibration curves of the nomogram model for predicting GBM patients’ 0.5-year **(F)**, 1-year **(G)**, 1.5-year **(H)**, 2-year **(I)**, 2.5-year **(J)**, and 3-year **(K)** survival.

Based on the TCGA dataset, the risk scores of clinical features (gender, MGMT. Promoter, age) and prognostic model were grouped for univariate cox and multivariate cox regression analysis respectively (**Table S3**). The results showed that the constructed prognostic model was an independent prognostic factor in the TCGA dataset (**Figure 11A**). The same analysis was conducted based on the verification set CGGA_325 (**Table S4**) and CGGA_693 (**Table S5**), and the results showed that the constructed prognostic model was also an independent prognostic factor in the validation set (**Figures 11B, C**). To further elucidate the predictive accuracy of the prognostic model and the above clinical features, we next performed an ROC analysis. The AUC of the risk score model was 0.5152 compared to 0.5088, 0.4767, and 0.5123 calculated for gender, MGMT. promoter status, and age respectively, which indicated that the risk score model can predict the OS of GBM patients with moderate sensitivity and specificity (**Figure 11D**). Then, we constructed a nomogram incorporating the abovementioned three clinical features and risk score to quantitatively estimate the 0.5-, 1-, 1.5-, 2-, 2.5-, and 3-year OS possibility of patients with GBM. As shown in **Figure 11E**, risk score contributed the most to prognosis, followed by age, gender and MGMT. promoter status. The total score was generated by summing the scores corresponding to each clinical feature to assess the probability of survival for each patient. The calibration curves also showed a favorable consistency between the nomogram predictions and actual observed outcomes of the 0.5-, 1-, 1.5-, 2-, 2.5-, and 3-year OS, indicating good prognostic accuracy of the model (**Figures 11F–K**).

### Evaluation of the function of CLEC5A *in vitro*

**Figure 12:**
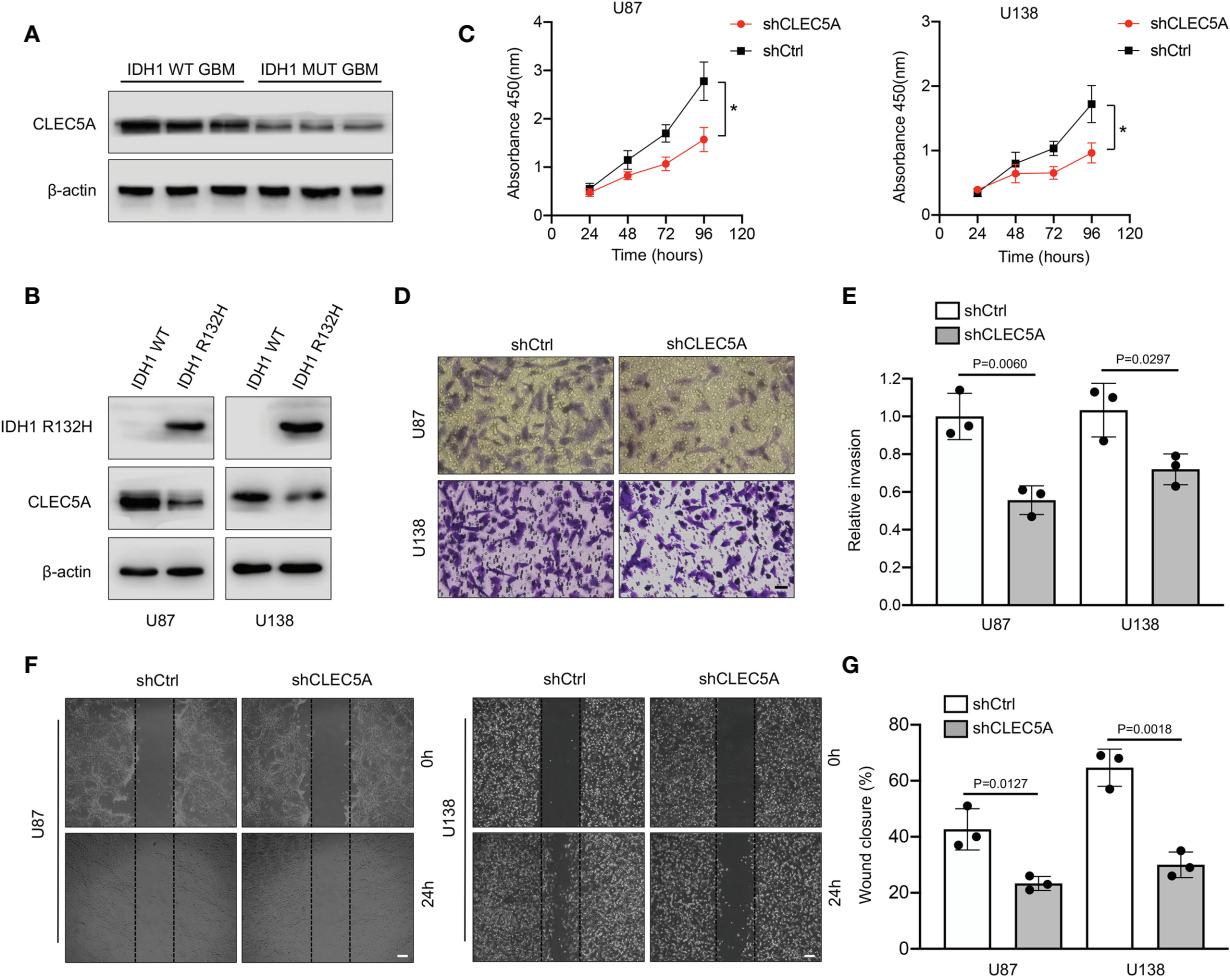
Evaluation of expression level and the function of CLEC5A *in vitro*. **(A)** Detection of the expression level of CLEC5A in IDH1 WT and MUT human GBM tissues by Western blotting. **(B)** Western blotting showing CLEC5A and IDH1 R132H protein levels in U87 and U138 GBM cells transduced with IDH1 R132H or vector control. **(C)** Percentage of viable U87 and U138 cells transfected with shCtrl or shCLEC5A. *p < 0.05. **(D, E)** Representative images of transwell invasion assays using U87 and U138 cells transfected with shCtrl or shCLEC5A. Quantification of transwell invasion assays is shown, scale bar = 100μm. **(F, G)** Wound healing assays using U87 and U138 cells transfected with shCtrl or shCLEC5A. Wound coverage was detected and photographed after 24h. Quantification of wound healing assays is shown, scale bar = 200μm.

The top two genes with the biggest regression coefficient in our constructed risk model were CLEC5A and MAPK8. Previous studies revealed that CLEC5A was included in a risk signature which served as an independent prognostic indicator for GBM (21, 22). In addition, a study by Fan et al (23). further demonstrated that CLEC5A participated in the regulation of PI3K/Akt signaling pathway and then promoted GBM malignant progression. Hence, we selected CLEC5A as a candidate gene and validated its function in GBM. We first tried to assess the expression level of CLEC5A in three IDH1 WT and three IDH1 MUT GBM specimens. As shown in **Figure 12A**, the expression level of CLEC5A in IDH1 WT GBM samples was significantly increased compared with that in IDH1 MUT GBM samples. To determine whether IDH1 R132H overexpression altered CLEC5A expression level, we overexpressed this protein in U87 and U138 cells. Results showed that CLEC5A protein level markedly decreased in IDH1 R132H-overexpressing GBM cells compared with corresponding control GBM cells (**Figure 12B**). Next, different experiments were performed to evaluate the change of GBM cell proliferation, invasion, and migration when CLEC5A expression was inhibited. Consistently with the experimental results of the previous study (23), silencing of CLEC5A remarkably retarded the growth of GBM cells (**Figure 12C**). Accumulating evidence demonstrated that the strong ability of tumor cells to invade adjacent and distant tissues constituted the major hindrances in GBM treatment (24). Hence, we next evaluated whether CLEC5A knockdown affected the invasion and migration of GBM cells. As shown in **Figures 12D, E**, depletion of CLEC5A significantly inhibited GBM cells invasion compared with the controls. Likewise, the wound coverage of the shCLEC5A-transfected GBM cells was also dramatically lower 24 h after plating, indicating that its migration ability was markedly decreased compared to the control group (**Figures 12F, G**). Taken together, these results suggest that CLEC5A showed differential expression level between IDH1 WT and MUT GBM and regulated GBM cell proliferation, invasion, and migration.

## Discussion

IDH1 is a key enzyme which can catalyze the oxidative decarboxylation of isocitrate to α-ketoglutarate (α-KG) in the Krebs cycle (5). It is generally accepted that IDH1 MUT GBM patients had better prognoses than IDH1 WT patients, but its specific mechanism is unclear. In GBM, it is proven that mutant IDH1 participates in a different metabolic pathway from wild type IDH1. The mutant IDH1 can catalyze α-KG into 2-HG, which has a similar molecular structure to α-KG. It means that 2-HG can competitively inhibit many α-KG dependent enzymes and then play an important role in the development of GBM (16, 25, 26). Although the effect of 2-HG on GBM is still controversial, the above conclusions all consistently suggest that IDH1 MUT GBM and IDH1 WT GBM have different metabolic modes, and their different prognosis may also be closely related to theifferrent metabolic modes. Therefore, finding the DEGs between IDH1 MUT and WT GBM, especially the metabolic related DEGs, will help to explain the reasons for the different prognosis between these two pathological types of gliomas.

Temozolomide (TMZ) is a kind of alkylating agent and currently recognized as a first-line chemotherapy drug for GBM (27). However, owing to the inherent or acquired resistance to TMZ, the overall effect of this drug in clinic is still unsatisfactory (28). Hence, exploration of new chemotherapy drugs which can be applied to GBM treatment is urgent. Study results from Peng et al. demonstrated that doxorubicin in combination with chemosensitizer lonidamine showed great anti-glioma efficacy *in vitro* and *in vivo* (29). A retrospective analysis (30) indicated that a combination agent with carboplatin and vinblastine shows similar efficacy compared with other single-agent and combination chemotherapy regimens in pediatric low-grade glioma. Furthermore, the chemotherapy agent sorafenib also exerted potent anti-glioma ability *via* inhibiting activation of multikinase (31). In the present study, we identified DEGs between IDH1 MUT and IDH1 WT GBM in several datasets, and confirmed six prognostic related GRGs which were significantly correlated with prognosis including CLEC5A, TNFAIP6, PLCB1, MAPK8, TMBIM1, and LDHA. Next, we incorporated these six hub genes and constructed a risk score model. Correlation analysis between the risk score and chemotherapy showed high-risk group GBM patients are more sensitive to doxorubicin and sorafenib compared with those in the low-risk group. In addition, stronger drug resistance of vinblastine was found in the high-risk group. Hence, our risk score model provides a potential theoretical basis for selection of chemotherapy drugs other than TMZ to treat GBM. This may open a new avenue for extension of GBM patient survival time, especially for those who show no response to TMZ. Among these six identified genes, PLCB1 plays critical roles in intracellular transduction and regulating signal activation, which are important to tumorigenesis (32). In GBM, PLCB1 was reported as one of the gene signatures related to chromosomal instability and phosphoinositide pathway (33). High TNFAIP6 expression is significantly positively associated with aggressive pathological characteristics, suggesting its roles in tumor development and progression (34, 35). However, the role of TNFAIP6 in GBM is still unknown and whether it has the same effect needs further study. Study results pointed out that MAPK8, a member of the JNK kinase family, was a protective factor for GBM patients’ survival (36). Loss of promotor methylation in glycolytic genes, especially LDHA, is associated with a more aggressive phenotype in IDH1 MUT GBM (37). Silencing of LDHA and downregulation of other glycolytic genes may help to explain the slower progression and better prognosis of IDH1 MUT GBM (37). TMBIM1 is demonstrated to attenuate GBM cell apoptosis and decrease the sensitivity of GBM cells to TMZ by inhibiting p38 phosphorylation (38). CLEC5A, encoding a C-type lectin, was found to be involved in GBM pathogenesis *via* regulation of the PI3K/Akt pathway (23). In addition, downregulation of CLEC5A can inhibit the capabilities of proliferation, migration, and invasion, and can promote apoptosis and G1 arrest in GBM cell lines (23). It was noteworthy that CLEC5A expression was higher in IDH1 WT GBM than IDH1 MUT GBM. Hence, we decided to validate its expression and function experimentally in GBM. Our results showed higher CLEC5A expression in IDH1 WT than MUT GBM and depletion of CLEC5A dramatically reduced GBM cell proliferation, invasion, and migration, further confirming its potential oncogenic role in GBM progression.

Immunotherapy has been proven to be effective in the treatment of multiple types of cancers, such as lymphoma (39) and melanoma (40). However, its efficacy for GBM was not ideal. The alterations in tumor metabolism and their subsequent influence on immune regulation has become increasingly recognized as important factors contributing to tumor growth and progression (41, 42). The shift to aerobic glycolysis in tumors has both active and passive consequences on the immune microenvironment (42–44). It was reported that IDH1 WT GBM exhibits more pronounced immunosuppressive characteristics than IDH1 MUT GBM, which may contribute to the different degrees of aggressiveness (45, 46). Thus, modulating the immunosuppressive microenvironment (ISME) is a promising strategy for improving the efficacy of immunotherapy. In this study, T cells CD8, plasma cells, T cells CD4 memory resting, T cell follicular helper, NK cells resting, NK cells activated, dendritic resting, dendritic cells activated, eosinophils, and neutrophils were significantly differentially expressed between high- and low-risk groups which were divided based on the score of our prognostic model.

The ISME has been demonstrated in the central nervous system tumors, especially for GBM (47). Some frontline immunotherapies were successfully applied in the clinic, which specifically target metabolic pathways and change the ISME. Also, metabolic pathways in GBM and their interactions with ISME and immune cells have the potential to exploit precise treatment approaches for GBM. For example, accumulation of regulatory T cells (Tregs) in the ISME resulted from high levels of co-stimulatory and co-inhibitory molecules expressed by effector CD8+ T-cells promote GBM progression through ameliorating auto-immunity (48). It is worth noting that 2-HG represents a unique metabolic pathway in a variety of tumors, including GBM. Mutations in the catalytic domains of IDH1 contribute to accumulation of 2-HG and modulation of anti-tumor immunity in GBM. Bunse et al. reported that 2-HG inhibits activity of critical enzymes or transcription factors such as ornithine decarboxylase, NF-kB p65, and NFATC1 so as to achieve the goal of impairing T cell function, especially CD4+ T cells (45). More importantly, IDH1 mutations also regulate anti-tumor immunity *via* decreasing PD-L1 expression and reducing immunosuppressive cell infiltration, indicating its potential possibility in GBM immunotherapy (49). Given the immunometabolic importance of IDH1 mutations, we establish a novel gene signature composed of several DEGs related to glycolysis between IDH MUT and IDH WT GBM. Although the association between the prognostic model and immunotherapy score is of no significance in the candidate IMvigor210 data set which contains immunotherapy information of urothelial carcinoma, the risk score model we construct could be utilized to predicate the potential clinical effects of immunotherapy for GBM patients based on the TIDE dataset. And, our results show that the risk signature significantly associated with patients’ outcome, clinical characteristics, tumor immunogenicity, immune infiltration, copy number variation, and immune matrix score, indicating its important role in immunity. Taken together, this gene model is not only able to predict patients’ prognosis, but also provide several possible gene targets of immunotherapy for IDH MUT and WT GBM based on their different immunometabolic pathways.

## Study limitations

Overall, this study utilized a bioinformatic approach to construct a risk model for prognosis prediction and risk stratification in the IDH1-associated GBM. However, there were several limitations in the present study. First, GS-MM correlation was not strong enough during determination of the key gene modules and hub genes. Perhaps a further analysis with a finer module splitting might help and we are eager to improve our methods in future. Second, the lack of six prognostic GRG-related specific GBM immunotherapy data sets forced us to adopt the IMvigor210 data set instead, which could perhaps bury differences in response to immunotherapy in high- and low-risk groups stratified by our model. In contrast, we used the TIDE dataset to evaluate the potential clinical effects of immunotherapy for GBM patients. The results indicated that patients in the high-risk group had a higher TIDE score compared with those in the low-risk group, suggesting a potential worse efficacy and more dismal outcome after acceptance of the immunotherapy treatment in the high-risk group than low-risk group, which confirmed a good prediction efficacy of our risk score model. Third, although *in vitro* functional studies of CLEC5A were performed here, an *in vivo* experiment was lacking. Further deep basic research is needed to verify the other five genes included in our risk model in future. Finally, we constructed a GRG-related prognostic signature to predict GBM patient survival by using TCGA dataset and validated our results through CGGA dataset and *in vitro* functional experiments in this study. Nonetheless, further validation in a larger GBM patient cohort is still warranted.

## Conclusion

In summary, we constructed and validated a novel risk score model of six prognostic GRGs based on the MSigDB, TCGA, and CGGA datasets for prognosis and risk stratification in IDH1 MUT and IDH1 WT GBM. Nomograms and ROC curves for 0.5-, 1-, 1.5-, 2-, 2.5-, and 3-year OS rate predictions were established and showed moderately excellent predictive efficacy in training and validation cohorts. Our established risk score model significantly associated with clinical characteristics, tumor immunogenicity, immune infiltration, copy number variation, and immune matrix score. And, the relationship between this model and immunotherapy was also demonstrated *via* the TIDE database. However, more deep basic research is needed to validate this prognostic model in IDH1-associated GBM.

## Data availability statement

The original contributions presented in the study are included in the article/**Supplementary material**. Further inquiries can be directed to the corresponding authors.

## Ethics statement

The studies involving human participants were reviewed and approved by Human Ethics Committee of Xinhua Hospital. The patients/participants provided their written informed consent to participate in this study.

## Author contributions

XC, SL, and MC conceived the project and designed the study. XC, ZC, CH, JS, WZ, and SF acquired, analyzed, and/or interpreted the data. XC and SF performed the experiments. XC wrote the manuscript. SL,YL, ZC, CH, and MC provided study supervision and/or revision of the article. All authors contributed to the article and approved the submitted version.

## Funding

This study was financially supported by the National Natural Science Foundation of China (No. 81902521), Shanghai Sailing Program (No. 19YF1432800), and the Research Project of Xinhua Hospital (No. XH1936).

## Conflict of interest

The authors declare that the research was conducted in the absence of any commercial or financial relationships that could be construed as a potential conflict of interest.

## Publisher’s note

All claims expressed in this article are solely those of the authors and do not necessarily represent those of their affiliated organizations, or those of the publisher, the editors and the reviewers. Any product that may be evaluated in this article, or claim that may be made by its manufacturer, is not guaranteed or endorsed by the publisher.

## Abbreviations

AUC, area under curve; CGGA, Chinese Glioma Genome Atlas; CLEC5A, C-type lectin domain containing 5A; DEG, differentially expressed genes; DMEM, Dulbecco’s modification of Eagle’s medium; FBS, fetal bovine serum; GBM, glioblastoma multiforme; GRG, glycolysis-related gene; IDH, isocitrate dehydrogenase; KEGG, Kyoto Encyclopedia of Genes and Genomes; LASSO, least absolute shrinkage and selection operator; LDHA, lactate dehydrogenase A; MAPK8, mitogen-activated protein kinase 8; PCA, principal component analysis; PLCB1, phospholipase C beta 1; ROC, receiver operational feature curve; shRNA, short hairpin RNA; TCGA, The Cancer Genome Atlas; TIDE, Tumor Immune Dysfunction and Exclusion; TNFAIP6, tumor necrosis factor alpha induced protein 6; TMBIM1, transmembrane BCL2-associated X inhibitor motif containing 1; WGCNA, weighted gene co-expression network analysis; WHO, World Health Organization.
