# Supplementary material for: Development of a novel glycolysis-related genes signature for isocitrate dehydrogenase 1-associated glioblastoma multiforme
Source: Front Immunol. 2022 Oct 28;13:950917. doi: 10.3389/fimmu.2022.950917 (PMC9650268; doi:10.3389/fimmu.2022.950917)
Supplement: Supplementary file 1 [file DataSheet_1.docx]

**Appendix S1**

**Development of a novel glycolysis-related genes signature for isocitrate dehydrogenase 1-associated glioblastoma multiforme**

Xiaomin Cai ^a,1^, Zheng Chen ^a,1^, Caiquan Huang ^b,1^, Jie Shen ^a,1^, Wenxian Zeng ^c, 1^, Shuang Feng ^d^, Yu Liu ^e,*^, Shiting Li ^f,*^, Ming Chen ^a,*^

**Supplementary Materials and Methods**

**Data preparation and collection**

The expression microarray data and clinical information of patients were collected from TCGA database through UCSC Xena website (http://xenabrowser.net/datapages/). The mutation data were downloaded by TCGA biolinks R package. The above data (409 samples) were all used as a training set. Likewise, 137 mRNAseq_325 and 237 mRNAseq_693 samples were collected from the CGGA (http://www.cgga.org.cn/). They were considered as a validation set. The immunotherapy data set for urothelial carcinoma (348 samples) was downloaded by IMvigor210 R package and used as a validation set for the prediction of GBM’s immunotherapy response. In addition, 200 glycolysis-related samples of genes set (HALLMARK_GLYCOLYSIS) were obtained from the Molecular Signature Database (MSigDB) (<http://www.gsea-msigdb.org/gsea/index.jsp>).

**Differential expression and functional enrichment analysis**

Limma R package was used for profiling analysis of differentially expressed genes (DEGs) between IDH1 MUT and IDH1 WT GBM samples. Any genes exhibiting the fold change (FC) ≥2.0, *p*-value<0.05 can be considered as significantly differential expression. The intersection genes between differentially expressed genes (IDH1 MUT vs IDH1 WT) and glycolysis-related genes were taken. The selected significant genes were further analyzed for Gene Ontology (GO) and Kyoto Encyclopedia of Genes and Genomes (KEGG) analysis by ClusterProfiler R package. *P*-value Cutoff = 0.05, p Adjust Method = “BH” and q value Cutoff = 0.2 were used as the parameters.

**Co-expression modules construction and hub genes identification**

Weighted gene co-expression network analysis (WGCNA) was used in this study to transform expression data into co-expression gene modules and explore the relationship between modules and phenotypic traits. The selected gene set was performed by “WGCNA” R package using the expression data of TCGA cohort. Scale independence and average connectivity degree of network with different power value were tested. The appropriate power value was determined when scale independence was above 0.85 with relatively higher connectivity degree. Then, according to topological overlap matrix (TOM) based dissimilarities, genes were sorted into different gene modules. The module with the highest correlation with clinical traits was regarded as key module and selected to identify candidate hub genes. Hub genes were determined by the overlap of candidate hub genes and genes selected from protein–protein interaction (PPI) network.

**Construction and validation of prognostic model**

The risk score was calculated by the following algorithm according to previous studies^1, 2^ (exp: expression value of gene for each patient, coef: the regression coefficient of gene in LASSO-Cox regression model):

Score=∑exp*coef

The samples were divided into high-risk and low-risk groups based on the median score. Kaplan-Meier univariate survival analysis was performed to generate the survival curve. The log-rank test was performed to validate the significance of differences (*p*-value <0.05).

To examine the prognostic accuracy of the score, we performed time-dependent receiver operating characteristic (ROC) analysis and calculated the area under the curve (AUC) by using “survival ROC” R package.

**Annotation of the immune infiltration microenvironment**

ESTIMATE was performed by “estimate” R package to evaluate the immune cell infiltration level (immune score) and stromal content (stromal score) for each sample. A leukocyte gene signature matrix consisting of 547 genes, which was termed LM22, was used to distinguish 22 immune cell types, and these types contained myeloid subsets, natural killer (NK) cells, plasma cells, naive and memory B cells and seven T cell types. We utilized CIBERSORT (https://cibersort.stanford.edu/) in combination with the LM22 signature matrix to estimate the fractions of 22 human hematopoietic cell phenotypes between IDH1 MUT and WT GBM. The sum of all estimated immune cell type fractions is equal to 1 for each sample.

**Prediction of immunotherapy response**

The IMvigor210 cohort, which is a urothelial carcinoma cohort treated with the anti-PD-L1 antibody atezolizumab was used for prediction of patient response to immunotherapy. The RNA-seq and clinical information of patients were collected from IMvigor210CoreBiologies. Fisher exact test was used to predict the responses of GBM patients to immune checkpoint inhibitors (ICIs).

**Cell culture**

The U87 and U138 GBM cell lines were purchased from the Cell Bank of the Chinese Academy of Sciences (Shanghai, China). All these cells were cultured at 37°C in 5% CO_2_ in Dulbecco's Modified Eagle Medium (DMEM, Life Technologies/Gibco, Carlsbad, CA, USA) supplemented with 10% fetal bovine serum (FBS, Life Technologies/Gibco, Carlsbad, CA, USA), 100 U/mL penicillin and 100 μg/mL streptomycin (Gibco, Grand Island, NY, USA).

**Lentiviral and plasmid transfection**

The lentivirus-based short hairpin RNA (shRNA) targeting CLEC5A, LV2-pGLVU6-shCLEC5A-Puro, was purchased from Genepharma (Shanghai, China). The target sequence was listed as follow: 5′–3′ CATTGGCCTAACAAAGACATT. To make lentivirus, the lenti-shCLEC5A and negative control were co-transfected with packaging plasmids into 293T cells following the manufacturer’s instructions. The viral‐containing media was harvested at 48 h post transfection, filtered and concentrated. To establish U87 and U138 cell stably expressing the wild-type and mutant IDH1, we purchased IDH1-R132H mutation lentivirus and wild-type lentivirus from GeneChem (Shanghai, China). For stable transfection, lentivirus was added to cells cultured in 6-well plates and selection with 5 mg/ml puromycin was performed 48 h after transfection. The cells were transfected using Lipofectamine 3000 Transfection Reagent (Invitrogen) according to the manufacturer’s instructions.

**Western blotting**

Briefly, cells were harvested and subjected to lysis using RIPA buffer containing protease and phosphatase inhibitors. Cell lysates were separated by SDS-PAGE followed by electrotransfering onto polyvinylidene difluoride membranes (PVDF) and were then incubated with the corresponding primary antibodies, washed, and probed with the indicated HRP-conjugated secondary antibodies. Antibody against CLEC5A was purchased from Abcam. Anti-IDH1 R132H antibody was obtained from Dianova. Anti-β-actin antibody purchased from Sigma-Aldrich, was used as the negative control.

**Cell proliferation assay**

Cell Counting Kit‐8 (CCK-8, Beyotime, Shanghai, China) was utilized to detect cell proliferation according to the manufacturer’s instructions. Brieﬂy, 3 × 10^3^ cells/well transfected with indicated plasmids were seeded into 96‐well plates and cultured. 10 uL CCK‐8 solution were added into each well at 24, 48, 72, and 96 h of culture. After 2‐hour incubation, the absorbance at 450 nm was detected using a microplate reader.

**Transwell invasion assay and wound healing assay**

The invasion assay was performed using 24-well Invasion Chambers pre-coated with Matrigel (BD Biosciences, USA). Approximately 5 × 10^4^ suitably treated GBM cells were seeded in the upper chamber with serum-free media. A chemo-attractant composed of DMEM containing 10% FBS was added to the lower chamber. After incubation for 24 hours, cells above the Matrigel layer were scraped off, and cells below the membrane were fixed by methanol, stained with 0.1% crystal violet for 10 minutes. The number of invading cells was counted in 5 randomly chosen fields ﬁelds per sample.

For wound healing assay, the suitably treated GBM cells were seeded into 6-well plates at the density of 3 × 10^5^ cells/well. Cell monolayers were scratched using a 200-μL plastic pipette tip when cell confluence reached 90% and then cultured in DMEM containing 1% FBS for 24 hours. The wound area was photographed at 0 and 24 h separately and wound closure was then calculated.

**References**

1 Chen X, Zhang D, Jiang F, et al. Prognostic Prediction Using a Stemness Index-Related Signature in a Cohort of Gastric Cancer. *Front Mol Biosci*. 2020; 7: 570702.

2 Shao W, Yang Z, Fu Y, et al. The Pyroptosis-Related Signature Predicts Prognosis and Indicates Immune Microenvironment Infiltration in Gastric Cancer. *Front Cell Dev Biol*. 2021; 9: 676485.
