# Supplementary material for: Development of a novel glycolysis-related genes signature for isocitrate dehydrogenase 1-associated glioblastoma multiforme
Source: Front Immunol. 2022 Oct 28;13:950917. doi: 10.3389/fimmu.2022.950917 (PMC9650268; doi:10.3389/fimmu.2022.950917)
Supplement: Supplementary file 2 [file DataSheet_2.docx]

**Development of a novel glycolysis-related genes signature for isocitrate dehydrogenase 1-associated glioblastoma multiforme**

Xiaomin Cai ^a,1^, Zheng Chen ^a,1^, Caiquan Huang ^b,1^, Jie Shen ^a,1^, Wenxian Zeng ^c, 1^, Shuang Feng ^d^, Yu Liu ^e,*^, Shiting Li ^f,*^, Ming Chen ^a,*^

^a^ Department of Neurosurgery, Xinhua Hospital, Shanghai Jiaotong University School of

Medicine, Shanghai, China.

^b^ Department of Neurosurgery, Sichuan Provincial People’s Hospital, University of Electronic Science and Technology of China, Chengdu, China.

^c^ Department of Neurosurgery, Zhujiang Hospital, Southern Medical University, Guangzhou, China.

^d^ Department of Encephalopathy, The Third Afﬁliated Hospital of Nanjing University of Chinese Medicine, Nanjing, China

^e^ Department of Neurosurgery, Shanghai Children’s Hospital, Shanghai Jiaotong University, Shanghai, China.

^f^ Department of Neurosurgery, Xinhua Hospital, Shanghai Jiaotong University School of Medicine, the Cranial Nerve Disease Center of Shanghai Jiaotong University, Shanghai, China.

^1^ These authors contributed equally to this work.

**^*^ Corresponding Authors:**

Ming Chen

Department of Neurosurgery, Xinhua Hospital of Shanghai Jiaotong University School of Medicine. 1665 Kong Jiang Road, Yangpu District, Shanghai, 200092, China.

E-mail: chenming@xinhuamed.com.cn

Shiting Li

Department of Neurosurgery, Xinhua Hospital of Shanghai Jiaotong University School of Medicine, the Cranial Nerve Disease Center of Shanghai Jiaotong University. 1665 Kong Jiang Road, Yangpu District, Shanghai, 200092, China.

E-mail: lishiting@xinhuamed.com.cn

Yu Liu

Department of Neurosurgery, Shanghai Children’s Hospital, Shanghai Jiao Tong University. 355 Lu Ding Road, Putuo District, Shanghai, 200062, China.

E-mail: liuyu@shchildren.com.cn

**Fig. S1**

**
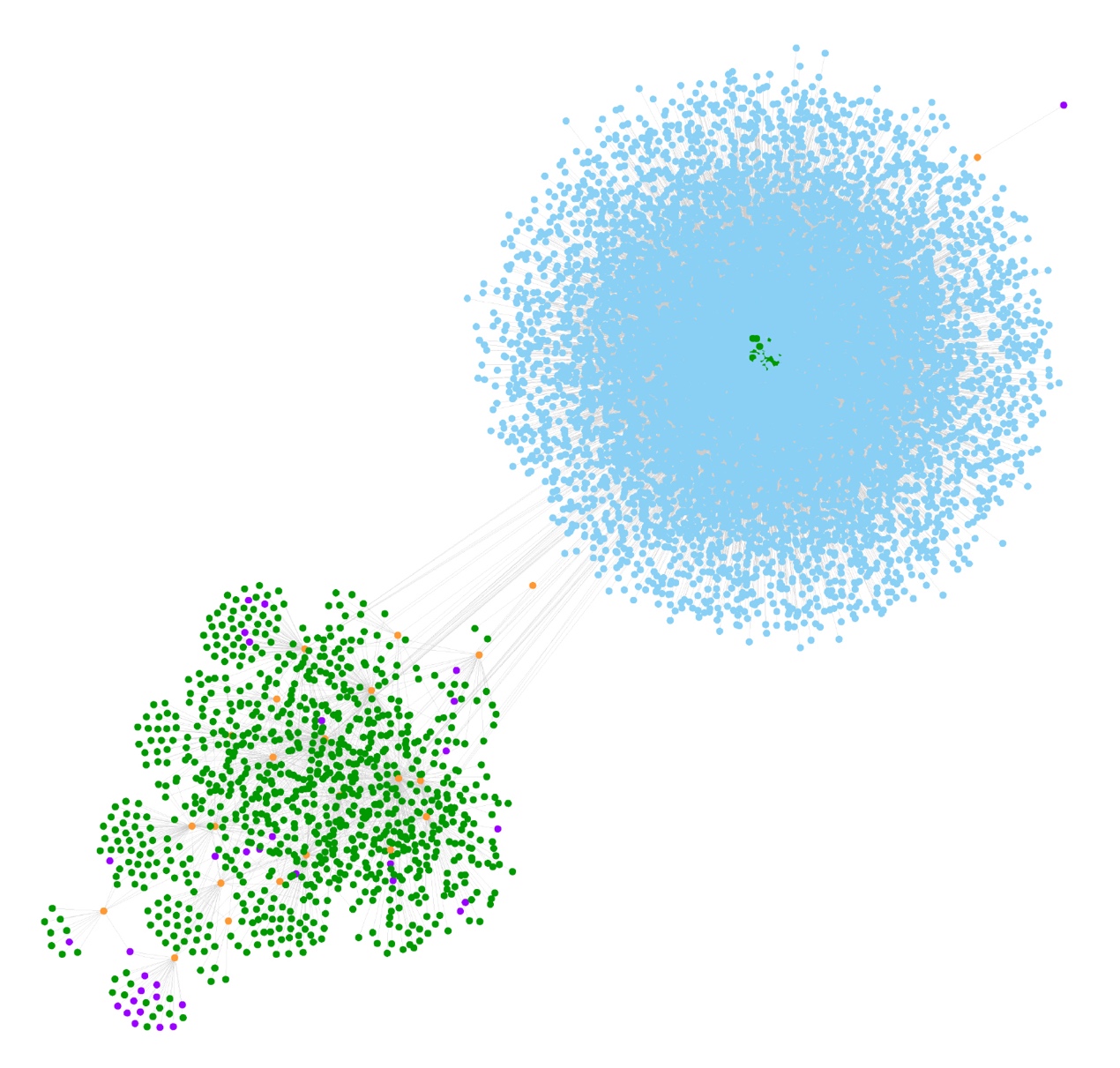
**

Map of the glycolytic core genes-related lncRNA-miRNA-mRNA-transcription factor network generated using TargetScan (http://www.targetscan.org/vert_72/), miRTarBase (<http://mirtarbase.mbc.nctu.edu.tw/php/index.php>), miRDB (http://mirdb.org/), and miRcode (http://www.mircode.org/). Glycolytic core genes are presented with yellow, lncRNA with blue, miRNA with green, and transcription factor with purple.

**Fig. S2**

**
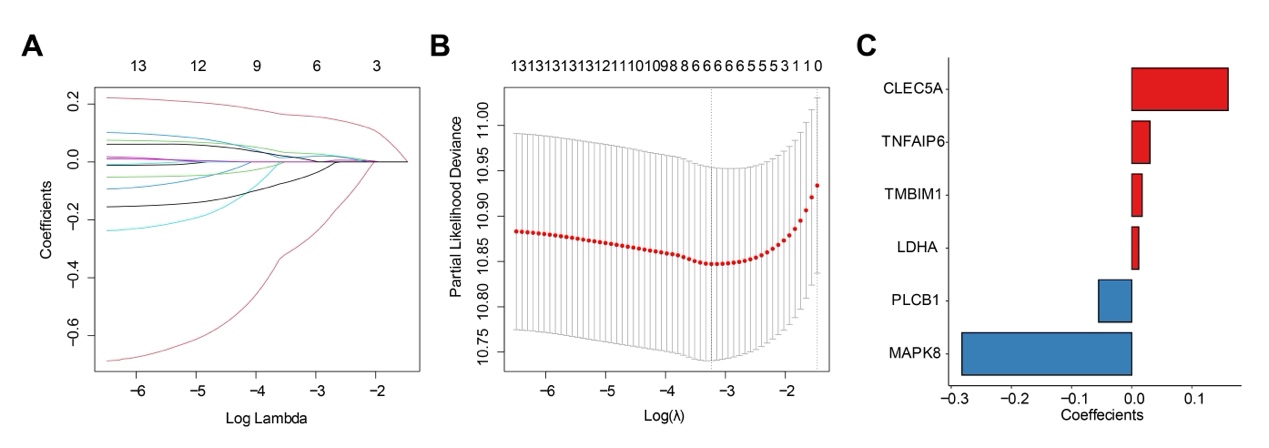
**

Construction of a risk score model using the LASSO analysis. A, B. LASSO coefficients profiles of 14 OS–associated glycolytic core genes. The partial likelihood deviance plot showed the minimum number corresponds to the covariates. C. LASSO coefficient configuration of the 6 key prognostic glycolytic core genes.

**Fig. S3**

**
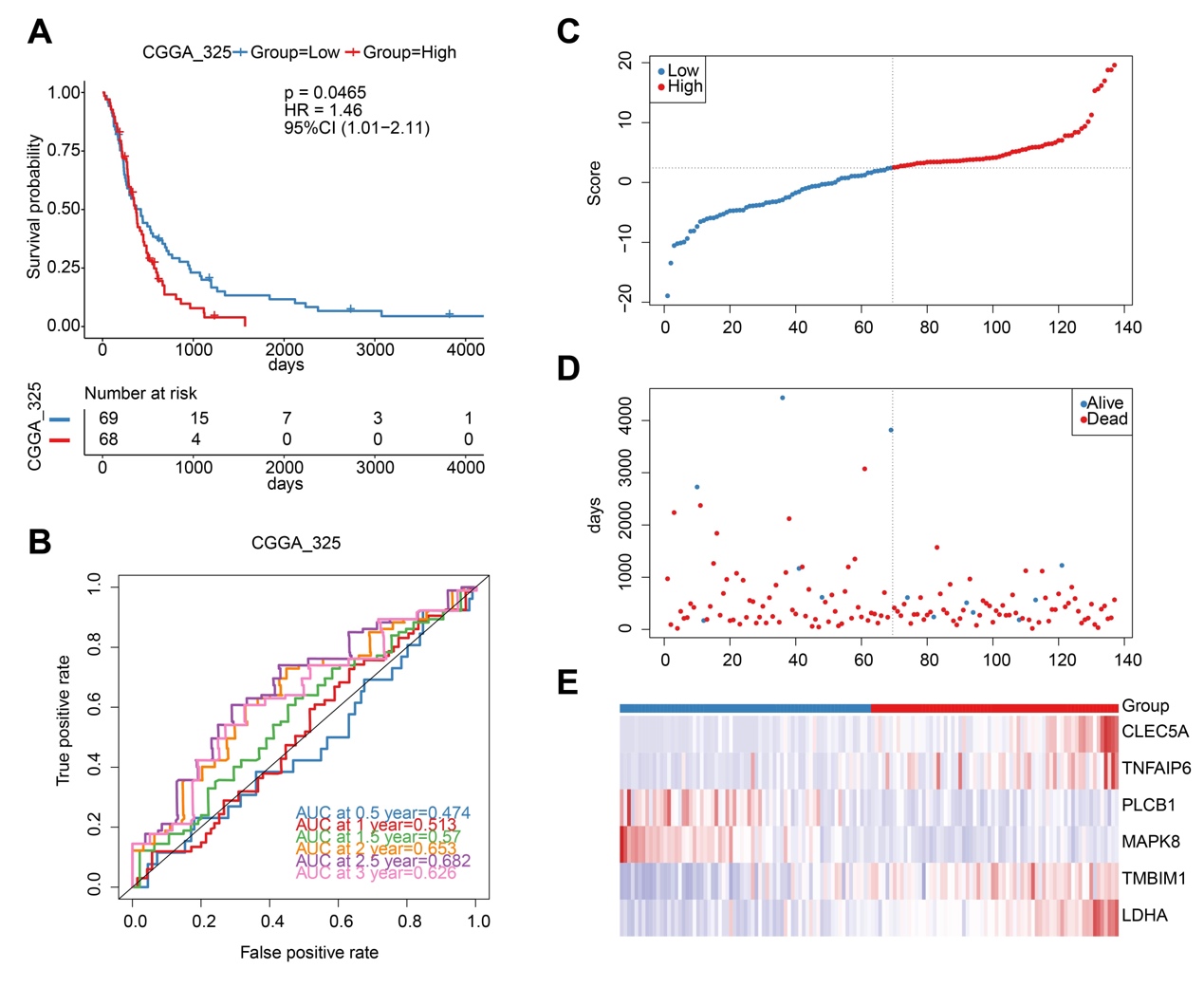
**

External verification of the risk score model in the CGGA_325 database. A. Kaplan-Meier survival curves for high- and low-risk groups stratified by the risk score model in the CGGA_325 database. B. ROC curves for predicting 0.5-year, 1-year, 1.5-year, 2-year, 2.5-year and 3-year overall survival for GBM patients based on the risk score in the CGGA_325 database. C-E. Risk score distribution, GBM patients' survival status and 6 key prognostic glycolytic core genes expression heat map in the CGGA_325 database.

**Fig. S4**

**
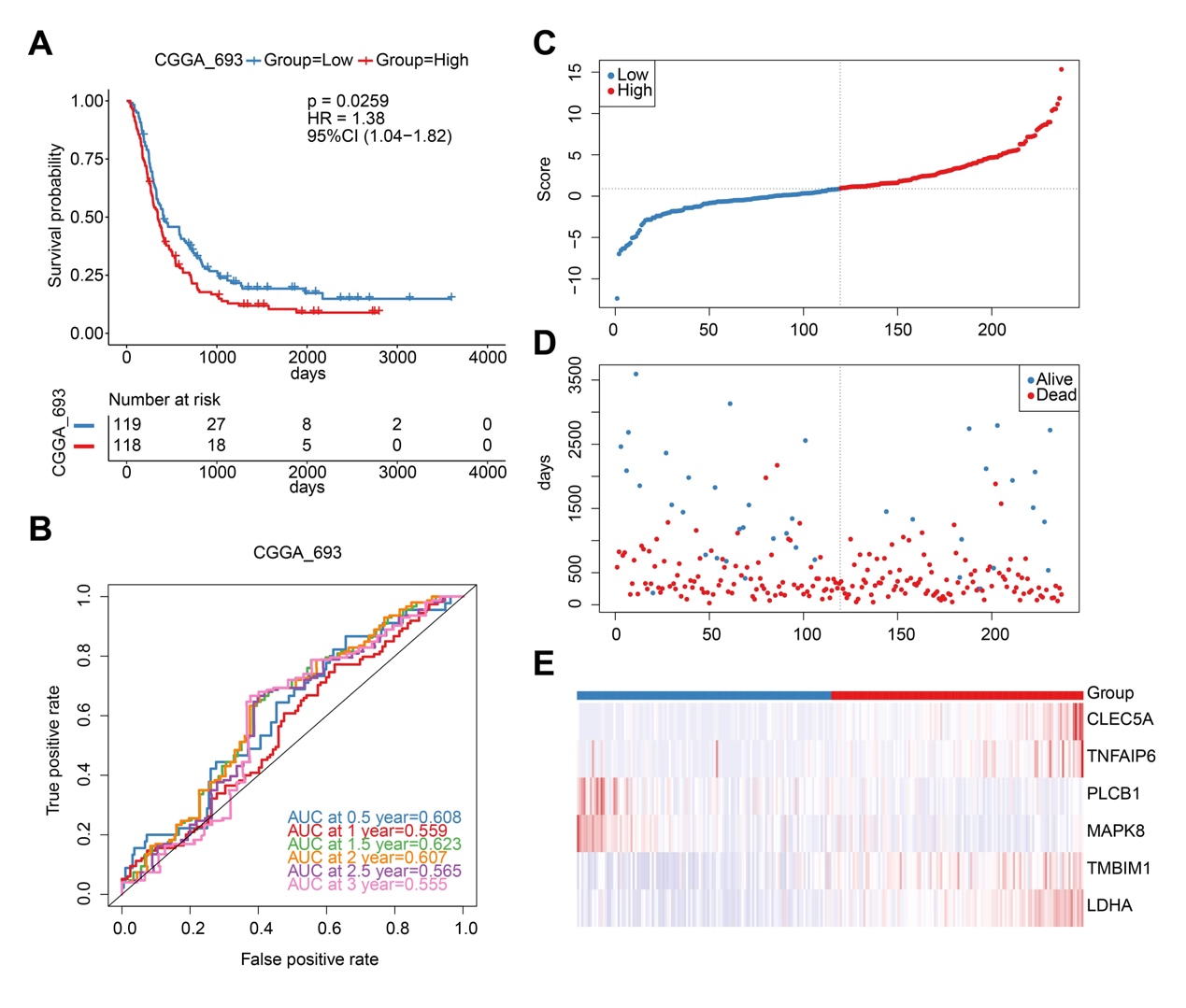
**

External verification of the risk score model in the CGGA_693 database. A. Kaplan-Meier survival curves for high- and low-risk groups stratified by the risk score model in the CGGA_693 database. B. ROC curves for predicting 0.5-year, 1-year, 1.5-year, 2-year, 2.5-year and 3-year overall survival for GBM patients based on the risk score in the CGGA_693 database. C-E. Risk score distribution, GBM patients' survival status and 6 key prognostic glycolytic core genes expression heat map in the CGGA_693 database.

**Fig. S5**

**
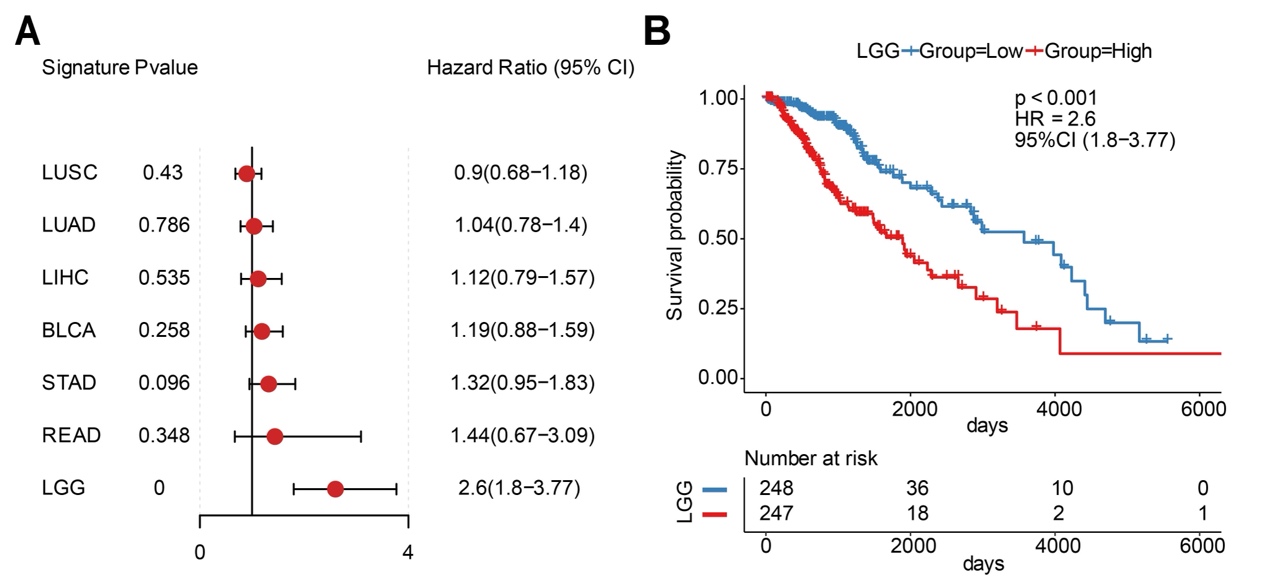
**

Validation of the prognostic model in other cancers of TCGA dataset. A. Pan-cancer Analysis of the prognostic model in other types of cancer of the TCGA dataset. B. Kaplan-Meier survival curves of LGG patients for high- and low-risk groups stratified by the prognostic model in the TCGA dataset.

**Fig. S6**

**
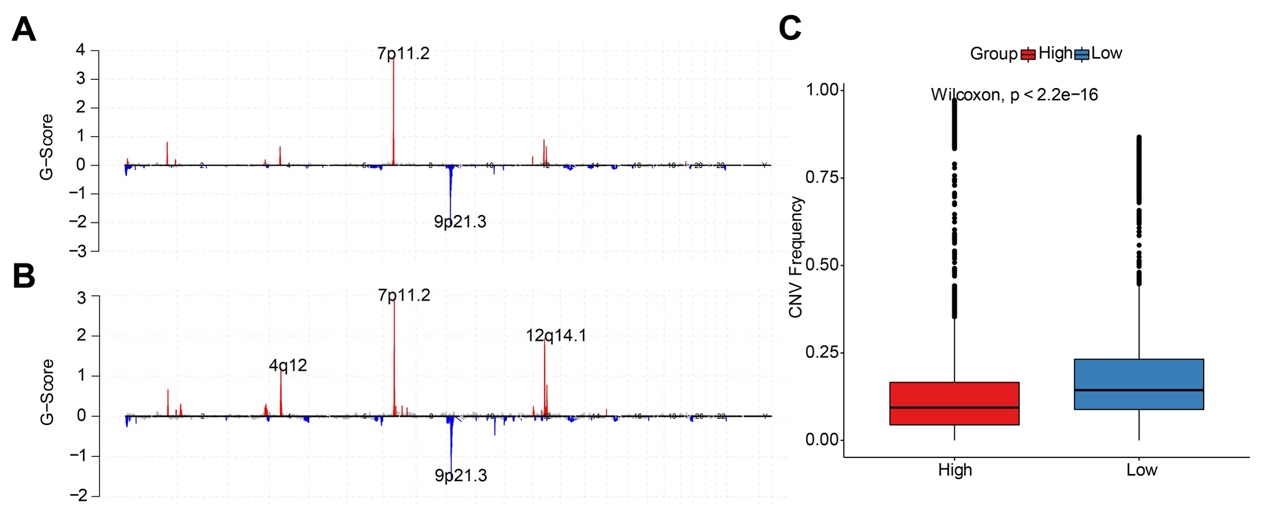
**

Comparison of copy number variation frequency between high- and low-risk groups. A, B. Distribution of the copy number variation region in high-risk group (A) and low-risk group (B). C. Copy number variation frequencies are significantly different in high- and low-risk groups.

**Fig. S7**

**
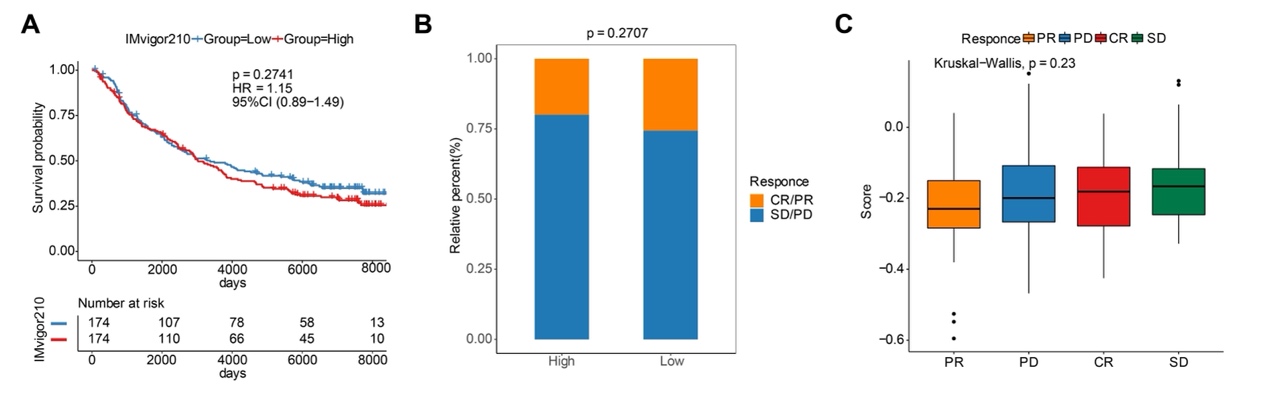
**

Relationship between risk score and immune response. A. Kaplan-Meier survival curves of GBM patients for high- and low-risk groups stratified by the prognostic model in the IMvigor210 dataset. B. Relative distribution of immune response in high-risk group and low-risk group. C. Risk score distribution of four different immune response samples.

**Fig. S8**

**
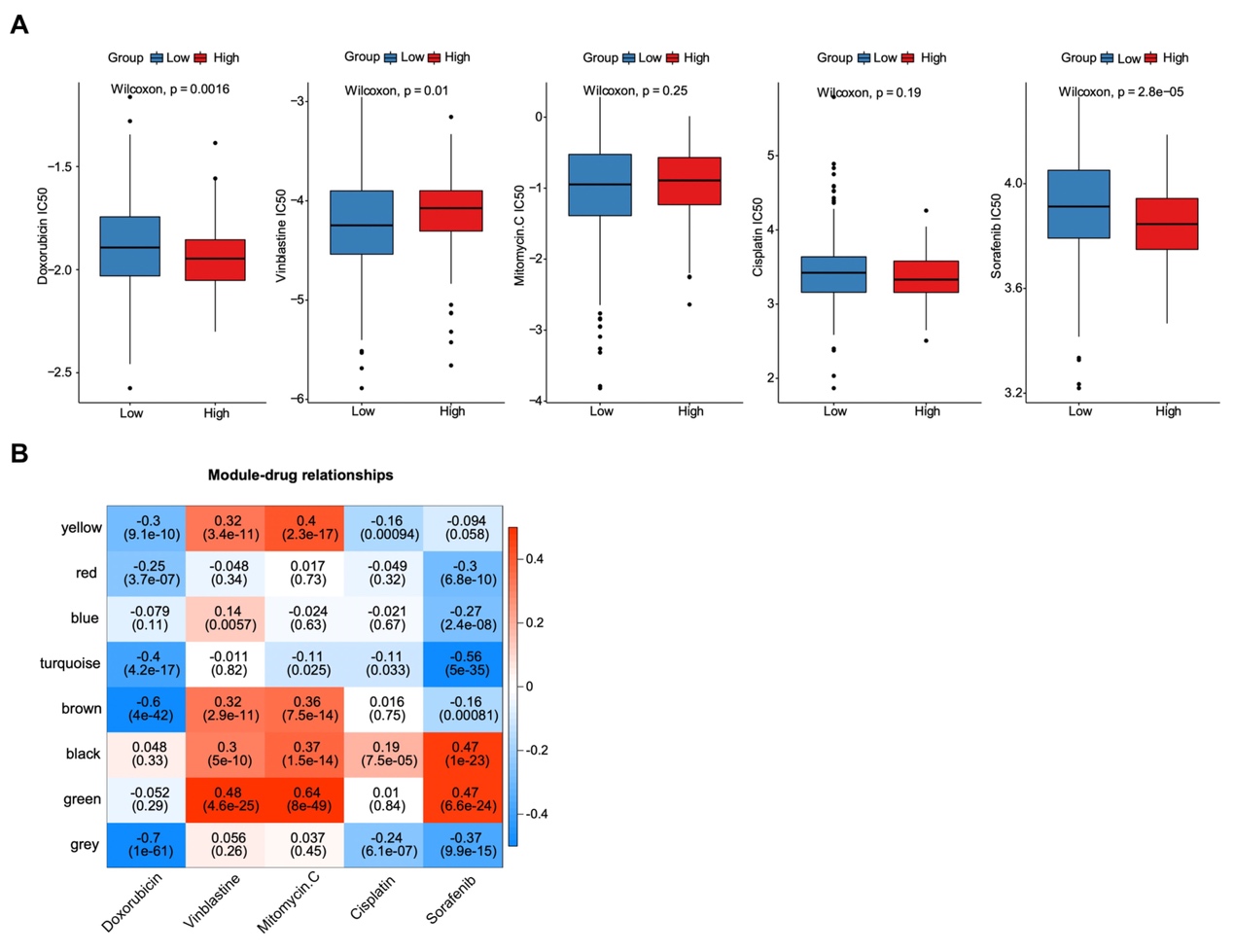
**

Resistance to 5 chemotherapeutic drugs in high-risk group and low-risk group and the relationship between gene modules and these 5 chemotherapeutic drugs. A. The half maximal inhibitory concentration (IC50) of different chemotherapeutic drugs is tested, including doxorubicin, vinblastine, mitomycin c, cisplatin, and sorafenib. B. The association between gene modules and different chemotherapeutic drugs.

**Fig. S9**

**
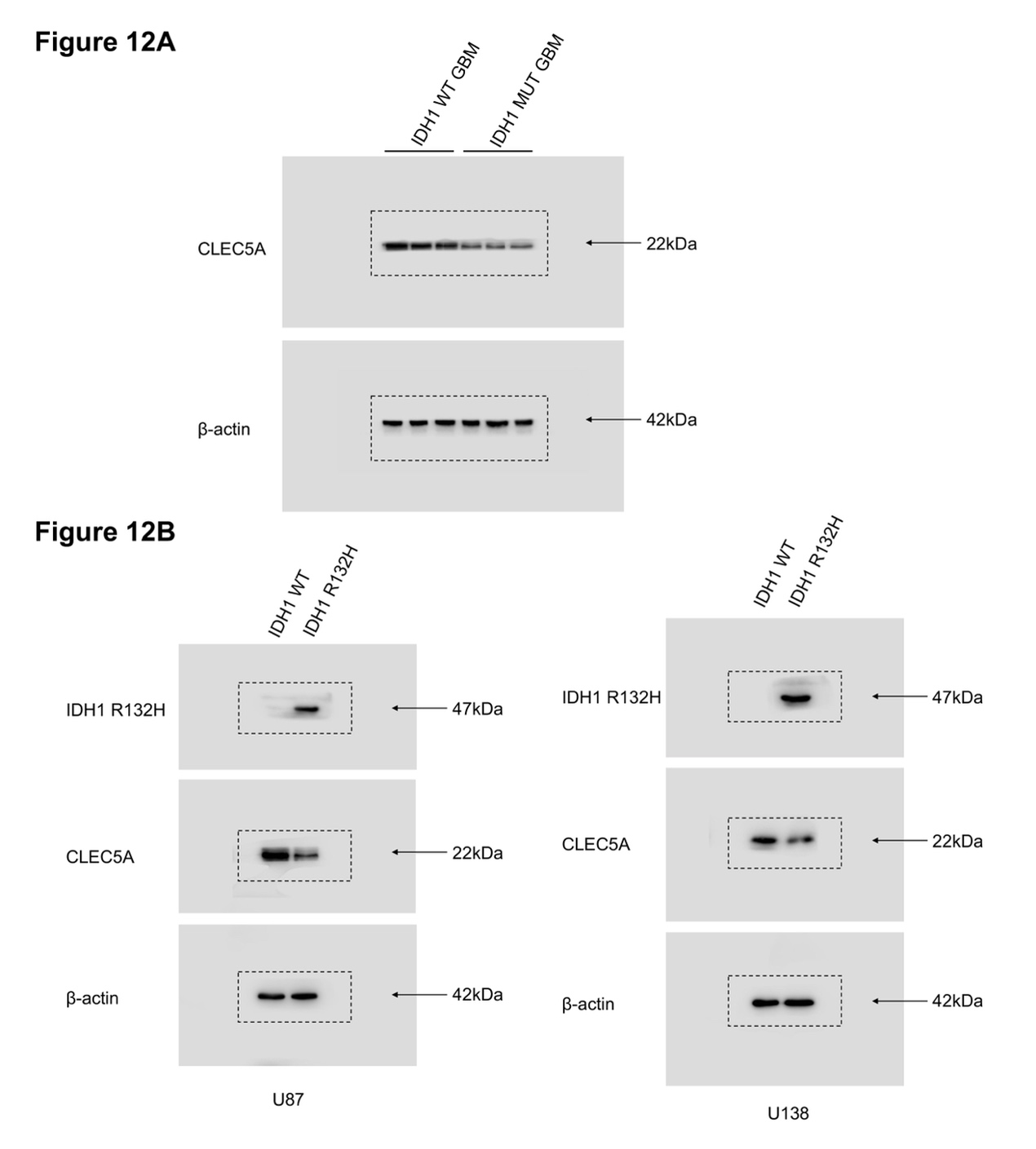
**

Uncropped images of Western blotting**.**
