# Supplementary material for: Development of a novel glycolysis-related genes signature for isocitrate dehydrogenase 1-associated glioblastoma multiforme
Source: Front Immunol. 2022 Oct 28;13:950917. doi: 10.3389/fimmu.2022.950917 (PMC9650268; doi:10.3389/fimmu.2022.950917)
Supplement: Supplementary file 3 [file Table_1.docx]

**Table S1.** Demographic and clinical characteristics of six GBM patients.

| Clinical characteristics | GBM1 | GBM2 | GBM3 | GBM4 | GBM5 | GBM6 |
| --- | --- | --- | --- | --- | --- | --- |
| Pathologic type | Glioblastoma | Glioblastoma | Glioblastoma | Glioblastoma | Glioblastoma | Glioblastoma |
| Primary or recurrent | Primary | Primary | Primary | Primary | Primary | Primary |
| Sex | Male | Female | Male | Male | Male | Male |
| Tumor location | Right temporal lobe | Right frontal lobe | Right insula lobe | Left frontal lobe | Right frontal lobe | Right frontal lobe |
| Age (years) | 68 | 77 | 36 | 62 | 47 | 35 |
| TMZ dose after surgery | 75mg/kg/m^2^ | 75mg/kg/m^2^ | / | 75mg/kg/m^2^ | 75mg/kg/m^2^ | 75mg/kg/m^2^ |
| MGMT. promotor mutation | Methylated | Methylated | Unmethylated | Unmethylated | Methylated | Unmethylated |
| IDH1 mutation status | Wildtype | Wildtype | Mutation | Wildtype | Mutation | Mutation |
| TERT. promoter mutation | Mutation | Mutation | Mutation | Mutation | Wildtype | Wildtype |
