# Supplementary material for: Development of a novel glycolysis-related genes signature for isocitrate dehydrogenase 1-associated glioblastoma multiforme
Source: Front Immunol. 2022 Oct 28;13:950917. doi: 10.3389/fimmu.2022.950917 (PMC9650268; doi:10.3389/fimmu.2022.950917)
Supplement: Supplementary file 4 [file Table_2.docx]

**Table S2.** Univariate cox regression analyses of 23 glycolytic core genes.

| Genes | Hazard ratio（95%CI） | P-value |
| --- | --- | --- |
| HRH1 | 1.32052 (1.05213 - 1.65738) | **0.01647** |
| LGALS1 | 1.16786 (0.93293 - 1.46196) | 0.17569 |
| CLEC5A | 1.84321 (1.4617 - 2.32431) | **0.00000** |
| TNFAIP6 | 1.38611 (1.10324 - 1.74151) | **0.00505** |
| GUSB | 1.27803 (1.01912 - 1.60273) | **0.03368** |
| TNFRSF1A | 1.40966 (1.12259 - 1.77013) | **0.00312** |
| TIMP1 | 1.43153 (1.14175 - 1.79485) | **0.00188** |
| SNAP91 | 0.88219 (0.70394 - 1.10556) | 0.27637 |
| HEXB | 1.3405 (1.06988 - 1.67958) | **0.01086** |
| PLCB1 | 0.7873 (0.62768 - 0.98751) | **0.03857** |
| HEBP1 | 1.11529 (0.89105 - 1.39595) | 0.34073 |
| ANXA2 | 1.18814 (0.94894 - 1.48764) | 0.13284 |
| MAPK8 | 0.72432 (0.57708 - 0.90912) | **0.00541** |
| ANXA1 | 1.21638 (0.96872 - 1.52735) | 0.09173 |
| SPRY2 | 1.3396 (1.06646 - 1.6827) | **0.01197** |
| TMBIM1 | 1.29597 (1.03413 - 1.6241) | **0.02436** |
| CHI3L1 | 1.33404 (1.06245 - 1.67504) | **0.01308** |
| SDC4 | 1.06596 (0.8514 - 1.33457) | 0.57751 |
| EPHB1 | 0.85215 (0.67957 - 1.06856) | 0.16585 |
| SH3GL2 | 1.12107 (0.89456 - 1.40493) | 0.32102 |
| PTX3 | 1.50794 (1.19765 - 1.89861) | **0.00048** |
| DNM3 | 0.97265 (0.776 - 1.21914) | 0.80987 |
| LDHA | 1.42712 (1.13573 - 1.79328) | **0.00227** |
