# Supplementary material for: Development of a novel glycolysis-related genes signature for isocitrate dehydrogenase 1-associated glioblastoma multiforme
Source: Front Immunol. 2022 Oct 28;13:950917. doi: 10.3389/fimmu.2022.950917 (PMC9650268; doi:10.3389/fimmu.2022.950917)
Supplement: Supplementary file 5 [file Table_3.docx]

**Table S3.** Univariate and multivariate cox regression analyses of clinical traits and risk score model in the TCGA dataset.

| Clinical traits | Univariate analysis |  | Multivariate analysis |  |
| --- | --- | --- | --- | --- |
|  | Hazard ratio（95%CI） | P-value | Hazard ratio（95%CI） | P-value |
| Age | 1.94(1.54-2.45) | 0.000 | 2.17(1.62-2.91) | 0.000 |
| Gender | 1.14(0.91-1.44) | 0.261 | 1.45(1.09-1.93) | 0.01 |
| MGMT. promotor | 0.72(0.55-0.95) | 0.02 | 0.8(0.6-1.05) | 0.11 |
| Risk model (Group) | 1.68(1.34-2.12) | 0.000 | 1.74(1.3-2.34) | 0.000 |
