# Supplementary material for: Development of a novel glycolysis-related genes signature for isocitrate dehydrogenase 1-associated glioblastoma multiforme
Source: Front Immunol. 2022 Oct 28;13:950917. doi: 10.3389/fimmu.2022.950917 (PMC9650268; doi:10.3389/fimmu.2022.950917)
Supplement: Supplementary file 6 [file Table_4.docx]

**Table S4.** Univariate and multivariate cox regression analyses of clinical traits and risk score model in the CGGA_325 dataset.

| Clinical traits | Univariate analysis |  | Multivariate analysis |  |
| --- | --- | --- | --- | --- |
|  | Hazard ratio（95%CI） | P-value | Hazard ratio（95%CI） | P-value |
| Age | 1.31(1.03-1.67) | 0.025 | 1.32(1.04-1.69) | 0.025 |
| Gender | 1.22(0.84-1.78) | 0.294 | 1.25(0.86-1.82) | 0.247 |
| MGMT. promotor | 0.9(0.55-0.95) | 0.553 |  |  |
| Risk model (Group) | 1.46(1.01-2.11) | 0.046 | 1.47(1.01-2.14) | 0.042 |
