# Supplementary material for: Development of a novel glycolysis-related genes signature for isocitrate dehydrogenase 1-associated glioblastoma multiforme
Source: Front Immunol. 2022 Oct 28;13:950917. doi: 10.3389/fimmu.2022.950917 (PMC9650268; doi:10.3389/fimmu.2022.950917)
Supplement: Supplementary file 7 [file Table_5.docx]

**Table S5.** Univariate and multivariate cox regression analyses of clinical traits and risk score model in the CGGA_693 dataset.

| Clinical traits | Univariate analysis |  | Multivariate analysis |  |
| --- | --- | --- | --- | --- |
|  | Hazard ratio（95%CI） | P-value | Hazard ratio（95%CI） | P-value |
| Age | 1.24(0.9-1.72) | 0.193 | 1.19(0.86-1.65) | 0.299 |
| Gender | 0.99(0.75-1.32) | 0.97 |  |  |
| MGMT. promotor | 0.92(0.68-1.25) | 0.6 |  |  |
| Risk model (Group) | 1.38(1.04-1.82) | 0.026 | 1.35(1.02-1.79) | 0.036 |
