# Supplementary material for: Development of a novel glycolysis-related genes signature for isocitrate dehydrogenase 1-associated glioblastoma multiforme
Source: Front Immunol. 2022 Oct 28;13:950917. doi: 10.3389/fimmu.2022.950917 (PMC9650268; doi:10.3389/fimmu.2022.950917)
Supplement: Supplementary file 8 [file Table_6.docx]

**Table S6.** Sources and versions of software and R packages used in this study.

| **R versions：R 4.1.2** | | | |
| --- | --- | --- | --- |
| **package_name** | **package_version** | **package_name** | **package_version** |
| magick | 2.7.3 | pRRophetic | 0.5 |
| cowplot | 1.1.1 | pROC | 1.18.0 |
| dendextend | 1.16.0 | ggpubr | 0.4.0 |
| ggdendro | 0.1.22 | rms | 6.2.0 |
| ggplot2 | 3.3.6 | clusterProfiler | 4.2.2 |
| survival | 3.2.13 | org.Hs.eg.db | 3.14.0 |
| survminer | 0.4.9 | reshape2 | 1.4.4 |
| survivalROC | 1.0.3 | limma | 3.50.0 |
| ComplexHeatmap | 2.10.0 | ggrepel | 0.9.1 |
| maftools | 2.10.0 | GSVA | 1.42.0 |
| plyr | 1.8.6 | tibble | 3.1.8 |
| ggplotify | 0.1.0 | tidyverse | 1.3.1 |
